# *OsTCP19* influences developmental and abiotic stress signaling by modulating ABI4-mediated pathways

**DOI:** 10.1038/srep09998

**Published:** 2015-04-29

**Authors:** Pradipto Mukhopadhyay, Akhilesh Kumar Tyagi, Akhilesh Kumar Tyagi

**Affiliations:** 1 National Institute of Plant Genome Research, Aruna Asaf Ali Marg, New Delhi. 110067, India

## Abstract

Class-I TCP transcription factors are plant-specific developmental regulators. In this study, the role of one such rice gene, *OsTCP19,* in water-deficit and salt stress response was explored. Besides a general upregulation by abiotic stresses, this transcript was more abundant in tolerant than sensitive rice genotypes during early hours of stress. Stress, tissue and genotype-dependent retention of a small in-frame intron in this transcript was also observed. Overexpression of *OsTCP19* in *Arabidopsis* caused upregulation of *IAA3*, *ABI3* and *ABI4* and downregulation of *LOX2,* and led to developmental abnormalities like fewer lateral root formation. Moreover, decrease in water loss and reactive oxygen species, and hyperaccumulation of lipid droplets in the transgenics contributed to better stress tolerance both during seedling establishment and in mature plants. OsTCP19 was also shown to directly regulate a rice triacylglycerol biosynthesis gene in transient assays. Genes similar to those up- or downregulated in the transgenics were accordingly found to coexpress positively and negatively with *OsTCP19* in Rice Oligonucleotide Array Database. Interactions of OsTCP19 with OsABI4 and OsULT1 further suggest its function in modulation of abscisic acid pathways and chromatin structure. Thus, *OsTCP19* appears to be an important node in cell signaling which crosslinks stress and developmental pathways.

Teosinte branched1, Cycloidea, Proliferating cell factor (TCP)-domain proteins are plant specific regulators of growth and organ patterning. These are basic helix-loop-helix (bHLH) transcription factors (TFs) but do not bind to E-Box DNA sequence. Sequence divergence in the TCP-domain of these non-conventional bHLH proteins further divides them into Class-I and -II TCP TFs, manifests position specific preferences for certain bases in their otherwise similar DNA-binding sequence and allows dimerization more freely between members of the same class^1^,^2^. The abundance of Class-I and -II TCP DNA-binding element in promoter of contrasting groups of genes creates functional antagonism between these two groups of proteins. While Class-I TCP TFs generally promote cell division and proliferation, and support the growth of organs and tissues, Class-II TCP proteins are known to function oppositely^3^. Also, owing to overlapping expression pattern and function of various Class-I TCP TFs, the phenotypes of their overexpression as well as mutant lines are mostly feeble or undetectable^4^,^5^.

In a wide variety of plants, TCP TFs regulate different developmental aspects through their effect on similar molecular pathways that include cytokinin, auxin, jasmonic acid (JA) and strigolactone^6^. These proteins also function by interacting with other TFs^5^,^7^ and regulate gene expression by recruiting chromatin modifiers like BRAHMA (BRM)^8^. TCP-regulated phenotypes include leaf shape, branch pattern, epidermal cell differentiation and floral structure and patterning^6^. TCP proteins have also been shown to integrate external signals into developmental pathways as exemplified by dark-responsive mesocotyl elongation in rice^9^.

The intrinsic developmental program of plants always remains knotted to external cues and is severely affected by abiotic stress conditions. Plants have developed mechanisms to withstand such harsh conditions by activating enzymes, transcription regulators and other factors that operate in pathways governed by hormones like abscisic acid (ABA) and second messengers like Ca^2+^. Interestingly, knockdown of a subset of Class-II TCP TFs by overexpression of *mir319* increases tolerance to dehydration and salinity stress in bentgrass^10^. Moreover, Ca^2+^-triggered signaling in *Arabidopsis* is known to activate genes through CAMTA-, DREB-, ABRE- and Class-I TCP-like factor binding sites in their promoter regions^11^. Mutation disrupting the function of *MSI1* (a transcriptional repressor), not only induces stress and ABA-responsive genes but also upregulates two Class-I TCP and a subset of Class-I TCP-regulated genes^12^. These reports do indicate a possible relation between pathways regulated by abiotic stress and ABA and those governed by Class-I TCP TFs.

In a previous study from our laboratory, based on microarray data, upregulation of *OsTCP19,* a Class-I TCP TF, in response to dehydration, salinity and cold was inferred^13^. The present work was undertaken to explore any possible role of Class-I TCP TFs in stress signaling network in rice. The results of the present work provide evidence about the possible mechanism by which OsTCP19 may confer salt and water-deficit tolerance.

## Results

### Abiotic stress-responsiveness of *OsTCP19*

**Figure 1:**
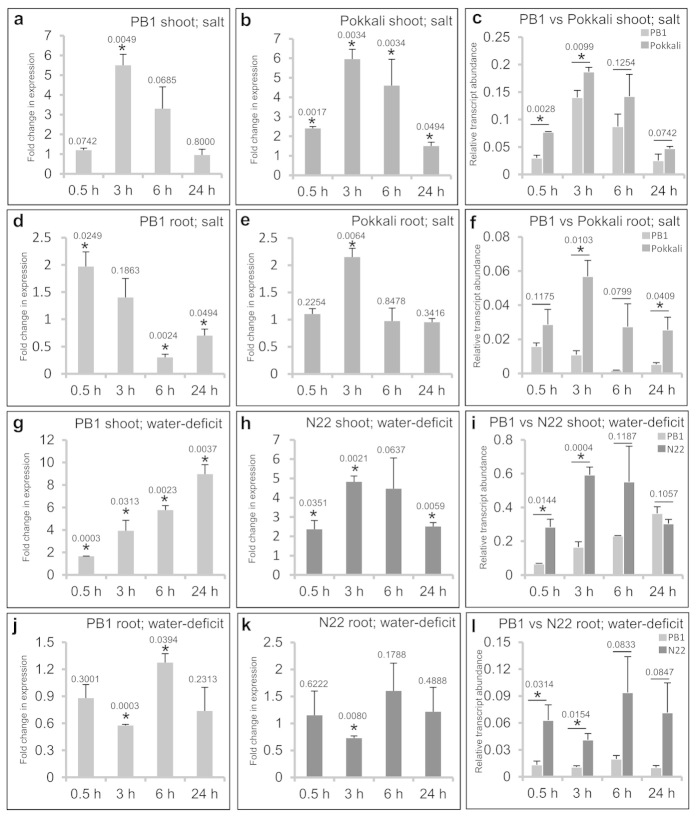
*OsTCP19* is upregulated under salt and water-deficit stress. **(**a,b,d,e,g,h,j,k) qRT PCR analysis indicating fold-change in expression (2^−ΔΔCt^ plot) of *OsTCP19* in shoots and roots of PB1, Pokkali and N22 rice under salt (200 mM NaCl) or water-deficit (air-drying) stress over their respective controls (unstressed tissue, 0 h sample). Value of control sample (not shown in the histogram) is equivalent to 1 and ‘*’ indicates data significantly different from control sample (*t*-test, two tailed p-value ≤ 0.05). (c,f,i,l) Comparison of the *OsTCP19* transcript level under water-deficit or salt stress in PB1, Pokkali and N22 rice varieties relative to the reference gene (*UBQ5*) expression (2^–ΔCt^ plot). ‘*’ indicates data significantly different from PB1 rice (*t*-test, two-tailed p-value ≤ 0.05). In all cases, X-axis indicates different time points after stress subjection and the error bars represent SD, and the p-value is mentioned over the respective bars. All data were simulated from three independent set of experiments (biological replicates). For each set of experiment, all varieties of rice were grown and subjected to stress simultaneously.

A previous microarray analysis from our laboratory pointed out an increase in expression of a Class-I TCP TF gene*, OsTCP19,* within a few hours exposure of rice seedlings to salt, drought and cold stress^13^ (GSE6901; Supplementary Fig. S1a,b online). To substantiate this observation and elucidate the role of this gene in stress tolerance, a detailed qRT-PCR analysis was conducted and the expression profile of *OsTCP19* from stress-sensitive indica rice variety Pusa Basmati 1(PB1) was compared with that from salt-tolerant Pokkali and drought-tolerant Nagina 22 (N22) rice genotypes under salt and drought stress, respectively. Compared to the untreated control samples (0 h), qRT-PCR analysis for shoots of 0, 0.5, 3, 6 and 24 h salt stressed PB1 and Pokkali rice seedlings confirmed 5 to 6-fold upregulation of this gene within 6 h of stress (Figure 1a,b). About 2-fold upregulation of this gene within 3 h of salt stress was also observed for roots of salt stressed PB1 and Pokkali seedlings (Figure 1d,e). While this expression increases up to 9-fold (by 24 h) and 5-fold (by 5 and 6 h) under water-deficit stress in shoots of PB1 and N22, respectively, water-deprived roots of both these varieties only show marginal fluctuation in transcript abundance (Figure 1g,h,j,k). Interestingly, a comparison of the relative transcript level with respect to the reference gene (*UBQ5*) expression (2^−Δ*Ct*^ plot) indicated higher abundance of *OsTCP19* in the tissues of stress-tolerant varieties than the sensitive PB1 variety at least during early hours of stress exposure (Figure 1c,f,i,l). These results suggest a probable role of *OsTCP19* in early response to abiotic stresses.

Expression of OsTCP19 was also positively influenced by exogenous application of stress-related hormones, namely, ABA, salicylic acid and methyl jasmonate (Supplementary Fig. S1c online). However, ABA caused most consistent and intensified expression (about 5-fold) of this gene, indicating a strong association of *OsTCP19* with ABA-mediated abiotic stress-signaling pathways. The untreated PB1 seedlings, incubated simply in fresh Yoshida medium for the same duration as mentioned for stress or hormone treatments, did not show any significant difference in *OsTCP19* expression (Supplementary Fig. 1d online). This indicated that the alteration in *OsTCP19* expression is specific to stress and hormone treatments.

### *OsTCP19* from indica rice contains an alternatively spliced intron

OsTCP19 was found to share more similarity with homologous protein sequences from monocots than other plant groups (Supplementary Fig. S2a online; Supplementary Table S1 online). Among *Arabidopsis* sequences, TCP15 and TCP14 were found closest (49–52% similarity) to OsTCP19 (Supplementary Fig. S2b,c online). RGAP database annotates this gene as intronless. However, its cloning using PB1 rice genomic DNA revealed the presence of an in-frame 36 bp insertion just before the designated TCP-domain. Owing to broad conservation in TCP-regulated pathways across plant species, this cloned fragment was overexpressed in *Arabidopsis thaliana* (Col-0) under the control of CaMV *35S* promoter (*p35S:OsTCP19*) for evaluating its role in stress tolerance (Supplementary Fig. S4a online). The 36 bp insertion and the flanking regions bear little similarity to *Arabidopsis* sequences. Hence, primers designed from the flanking regions (36-i primers) were used in a RT-PCR analysis meant for recording the level of expression of the transgene. This, however, resulted in amplification of about 136 bp DNA fragment instead of 172 bp suggesting this insertion, which begins and ends with GC and AG dinucleotides, is spliced and represents an intron.

**Figure 2:**
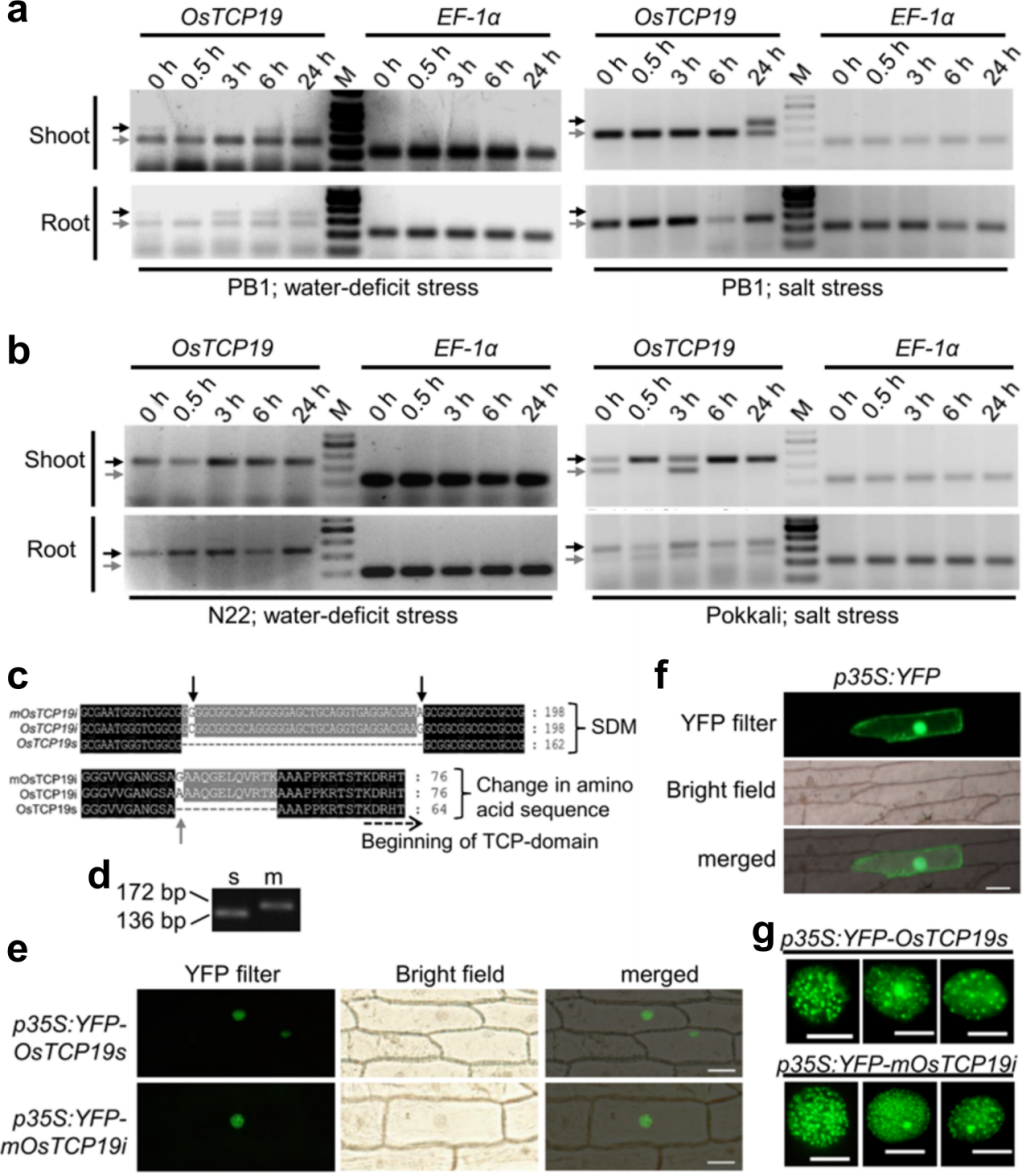
Splicing of *OsTCP19* and subcellular localization of the encoded proteins. (a,b) RT-PCR analysis using primers flanking an intron of *OsTCP19* and *OsEF1α* for unstressed (0 h) and stressed (0.5-24 h) tissues of indica rice seedlings (as indicated). M indicates the marker lane. The black and grey arrows correspond to band size of 172 bp and 136 bp, respectively. (c) ClustalW alignment showing region of mutation (black arrows) at the intron boundaries of *mOsTCP19i* relative to *OsTCP19s* and *OsTCP19i*, and the corresponding change in protein sequence (grey arrow). The numbers correspond to the respective nucleotide or amino acid position. (d) RT-PCR analysis of tobacco leaves infiltrated with *Agrobacterium* cells bearing construct *p35S:OsTCP19s* (lane s) and *p35S:mOsTCP19i* (lane m) using primers flanking OsTCP19 intron. (e,f) Fluorescence microscopy of onion epidermal cells expressing either *YFP*, *YFP-mOsTCP19i* or *YFP-OsTCP19s* as indicated (scale bar = 50 µm). (g) Higher resolution images of nuclear YFP fluorescence (scale bar = 10 µm).

Further RT-PCR analyses for studying the splicing of this gene using 36-i primers detected higher abundance of the spliced form (*OsTCP19s*; 136 bp amplicon) than the unspliced form (*OsTCP19i*; 172 bp amplicon) of *OsTCP19* in all tested samples of PB1 rice except 24 h salt stressed shoots and 3 h or more water-deficit stressed roots (Figure 2a). As amino acid stretch similar to that encoded by the 36 bp intron is present in homologous proteins from other monocots (Supplementary Fig. S2d online), higher abundance of *OsTCP19i* in other rice varieties appeared possible. On further analysis, *OsTCP19i* was observed as the major transcript form in all the stressed tissues of N22 whereas *OsTCP19s* transcript seems scarcely detectable (Figure 2b). While both forms were detected in unstressed Pokkali shoots, *OsTCP19i* was significantly enriched under salt stress (Figure 2b). Expression of *OsTCP19s* remained rather low in Pokkali roots. Any possibility of genomic DNA contamination in these assays was ruled out by a control RT-PCR using primers flanking an intron of *OsEF1α* (*LOC_Os03g08020*) which only amplified DNA fragment (103 bp) of size expected from cDNA. A graphical representation of this analysis is shown in ‘Supplementary Fig. S3 online’. Thus, it appears that *OsTCP19* from indica rice bears an alternatively spliced GC-AG intron and its splicing is dependent on plant type, variety, tissue and stress condition.

For further characterization, *OsTCP19s* was cloned from PB1 rice seedlings. The 5’ and 3’ intron boundaries of OsTCP19i were also mutated (5’GC≫GG, 3’AG≫AA) to restrict its splicing (*mOsTCP19i*) which, however, resulted in an Ala to Gly transition in the protein sequence (Figure 2c). This mutant construct was validated experimentally and only unspliced transcripts could be detected in tobacco leaf cells transiently expressing mOsTCP19i under the regulation of CaMV 35S promoter (Figure 2d). Particle bombardment of constructs bearing these ORFs fused to C-terminus of *YFP* (*p35S:YFP-mOsTCP19i* and *p35S:YFP-OsTCP19s)* on onion epidermal cells revealed the nuclear enrichment for both the proteins (Figure 2e). Control construct, *p35S:YFP*, generates fluorescence dispersed throughout the cell (Figure 2f). In addition to their presence in the whole nucleus, both these proteins were also detected in the form of multiple nuclear bodies (NB; Figure 2g) indicating the role of OsTCP19 in transcription as well as other nuclear phenomena.

### Phenotype and stress response of *p35S:OsTCP19* *Arabidopsis* transgenics

**Figure 3:**
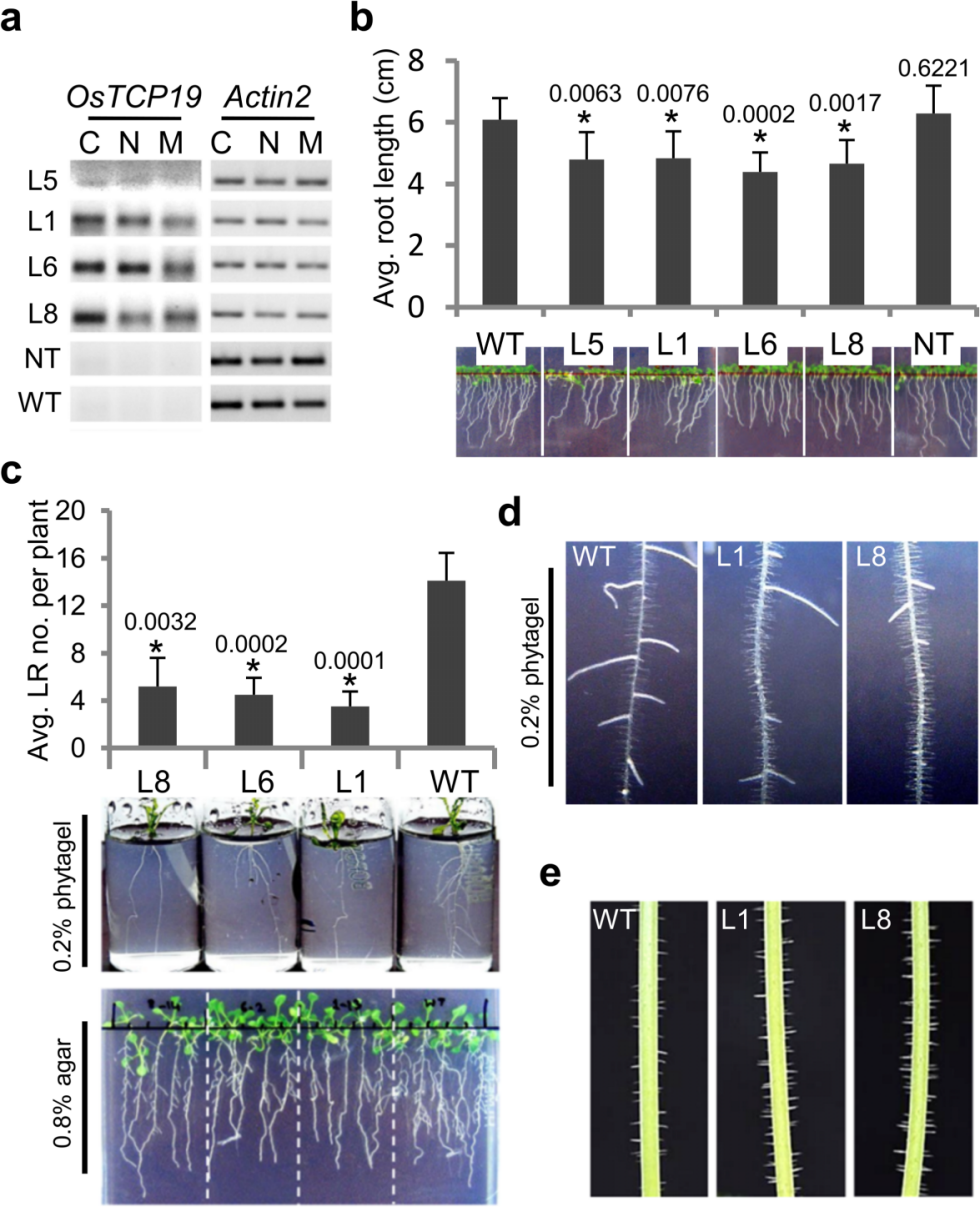
Phenotypes of *p35S:OsTCP19 Arabidopsis* transgenic plants. (a) Semi-qRT-PCR using 36-i primers depicting amplification of 136 bp fragments in transgenics (L1, L5, L6, L8) but not in NT and WT plants under control (C; unstressed), salt stress (N; 125 mM NaCl) and water-deficit stress (M; 350 mM mannitol). *ACT2* was used as endogenous control. (b) Root growth in 10-day-old transgenic, NT and WT seedlings. The analysis was performed with a total of 250–300 plants grown in three independent batches. (c) LR formation in 15-day-old transgenic and WT seedlings grown in glass vials containing 0.2% phytagel or Petri plates containing 0.8% agar as solidification base. Histogram was plotted from the analysis of at least 100 plants grown in three independent batches in Petri plates. (d) RH in 15-day-old seedlings of transgenic and WT plants. (e) Trichomes in ~11 cm long inflorescence stem of transgenic and WT plants. Error bars in the histograms indicate SD. ‘*’ indicates data significantly different from WT (*t*-test, two-tailed p-value ≤ 0.05). In all histograms, the p-value is mentioned over the respective bars.

For functional analysis, four T3 generation *p35S:OsTCP19* transgenic lines, L1, L5, L6 and L8, homozygous for single insertion were selected. During screening of T0 seeds, a line negative for hygromycin selection marker was also picked. This line, NT, was used as a negative control besides wild-type, WT, plants in various analyses. As mentioned before, *OsTCP19s* but not *OsTCP19i* transcripts could be detected by RT-PCR analysis in the transgenic seedlings grown on only MS medium (control) or supplemented with 125 mM NaCl or 350 mM mannitol (Figure 3a). Moreover, the expression was rather low in L5 compared to other transgenic lines, whereas no specific amplification was obtained for WT and NT plants.

Although both transgenic and non-transgenic plants displayed similar efficiency and rate of germination (Supplementary Fig. S4b online), slower initial growth of the transgenic lines was clearly inferred and was evident by reduced rate of root elongation till 15 days after germination (DAG; Figure 3b, Supplementary Fig. S4e online). Strikingly, by 15 DAG, the transgenic plants displayed significantly fewer numbers of lateral roots (LRs) as compared to WT plants (Figure 3c). The transgenic plants visually appeared to have higher number of trichomes on the inflorescence stem and more root hairs (RHs; Figure 3d,e), indicating a possible role of *OsTCP19* in epidermal cell differentiation as well. In addition, early flowering was observed in the transgenic lines grown on MS-agar medium inside vertically oriented Petri plates (Supplementary Fig. S4c online). However, this phenotype could not be inferred convincingly in plants grown in pots containing Soilrite mix. Therefore, constitutive overexpression of *OsTCP19* in *Arabidopsis* affects initial seedling growth, LR development, trichome and RH formation, and condition-dependent early flowering.

**Figure 4:**
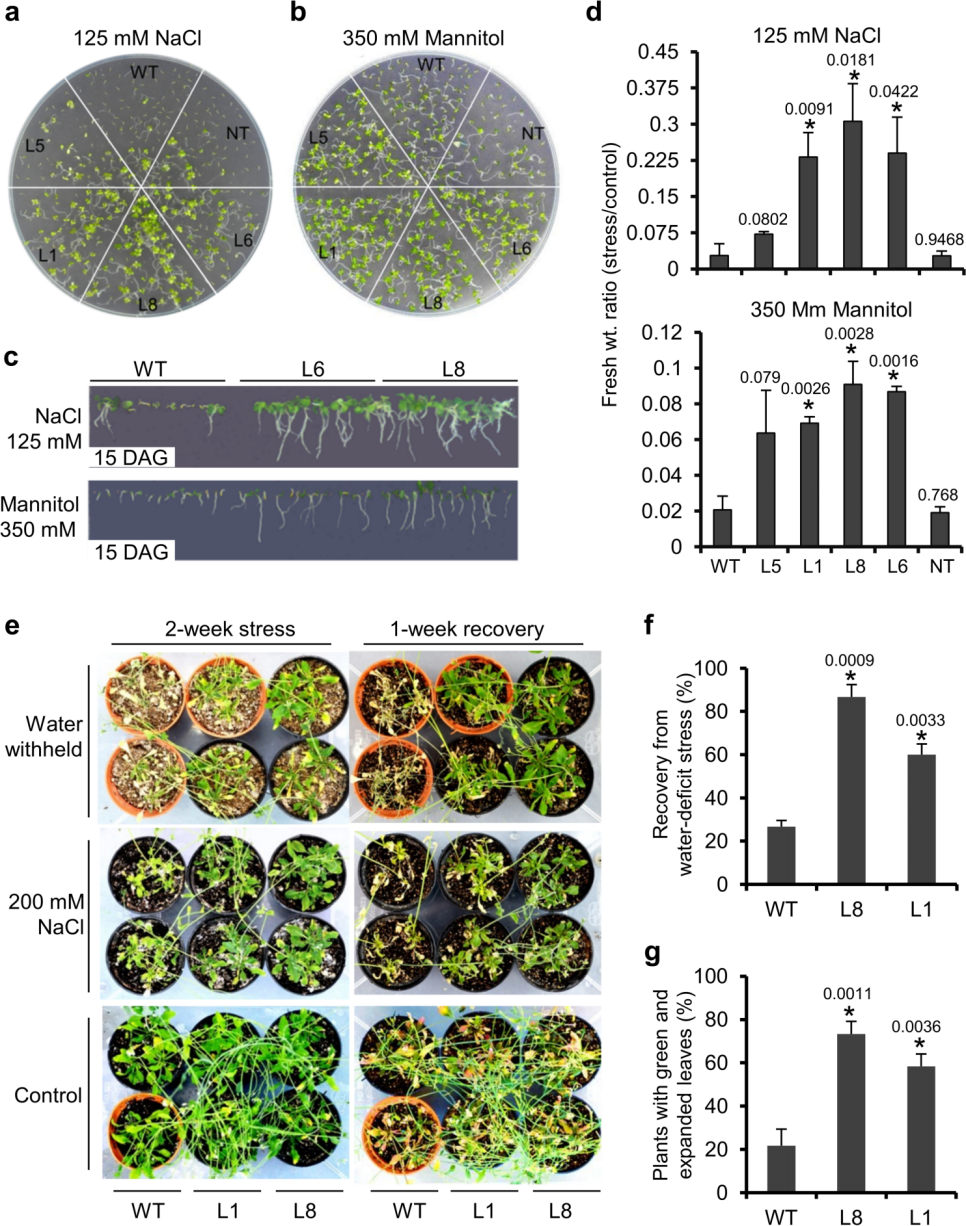
Abiotic stress tolerance of *p35S:OsTCP19 Arabidopsis* transgenic plants. (a–c) Post-germination growth and seedling establishment of WT, NT and transgenic plants (15 DAG) in response to salt and water-deficit stress under horizontal and vertical growth conditions. (d) Ratio of total biomass accumulated (per 100 seeds sown) by different transgenic, WT or NT lines under abiotic stresses (as indicated) to that under control condition. (e) Analysis of water-deficit (water withholding) and salt (200 mM NaCl) tolerance level in 2-week stresses plants (24-day-old) that were allowed to recover for 1 week. (f,g) Percentage of plant recovered or survived at the end of recovery phase. Error bars in the histograms represent SD. ‘*’ indicates data significantly different from WT (*t*-test, two-tailed p-value ≤ 0.05). The p-value is mentioned over the respective bars of the histograms. All analyses were performed with plants grown in three independent batches (biological replicates). For each independent set of experiment, the data was either simulated from six Petri plates (for a–d) or 50 plants (for e–g).

The seeds of WT, NT and transgenic plants had no major difference in germination response under salt and water-deficit stress (Supplementary Fig. S4f online). However, following germination, seedling establishment, growth and biomass accumulation were strikingly better in transgenic lines than WT or NT plants under higher (125 mM NaCl or 350 mM mannitol) but not lower (100 mM NaCl or 200 mM mannitol) degree of stress (Figure 4a–d; Supplementary Fig. S4g,h online). These results suggest a role for *OsTCP19* in stress-responsive post-germination growth, seedling establishment and biomass accumulation but not in germination *per se*. In a different experiment, when 12-day-old unstressed plants were transferred and observed for further 33 days in vertically oriented Petri plates containing MS-agar medium supplemented with 100 mM NaCl, better efficiency of flowering was found in transgenic than WT plants (Supplementary Fig. S4d online). This probably is related to mechanisms that caused early flowering in unstressed plants (Supplementary Fig. S4c online).

Compared to WT, detached leaves of 22-day-old plants of transgenic lines L1 and L8 suffered less cell death after 15 h incubation in salt solution which was evident by weaker staining using Evans blue (Supplementary Fig. S5a online). Decrease in the rate of water loss in detached leaves of transgenic plants was also clearly revealed by monitoring the time-dependent loss in fresh weight (Supplementary Fig. S5b online). When stressed with 200 mM NaCl or by water-withholding for two weeks, the 24-day-old L1 and L8 transgenic lines displayed better survival and appeared healthier than WT plants (Figure 4e). Not only better relative water content (RWC) but also reduced reactive oxygen species (ROS) accumulation, as revealed by H_2_DCFDA staining, was observed in the leaves of 12-day stressed transgenic plants (Supplementary Fig. S5c-e online). Within 1-week of irrigation with RO water, while nearly 80% L8 and 60% L1 transgenics recovered from water-deficit stress, this recovery was confined only to 25–30% WT plants (Figure 4e,f). None of the tested plant lines showed recovery from salt stress during 1-week of irrigation with RO water. Nonetheless, the transgenics displayed a slower rate of death since significantly more number of transgenics bearing green and expanded leaves was observed towards the end of this recovery phase than the WT plants (Figure 4e,g). Plants grown only under control condition do not display any distinct difference between them (Figure 4e).

### Overexpression of *OsTCP19* affects ABA, auxin and JA signaling in *Arabidopsis*

Since *OsTCP19* overexpression transgenics were compromised in LR development, it was decided to explore the underlying changes in gene expression to gain knowledge about the role of *OsTCP19* in the signaling network**.** Moreover, as TCP TFs influence the expression of genes of various hormonal pathways, important regulators of LR development belonging to such pathways were picked for expression analysis in the transgenics. Inhibition of various auxin responsive factors (ARFs) by many AUX/IAAs and modulation of PIN transporters are known to cause reduction in LR formation^14^. Elevation of endogenous cytokinin level and inhibition of jasmonate signaling also attenuates LR formation^15^,^16^. Consistent with these reports, genes for four AUX/IAAs (*IAA3*, *IAA12*, *IAA14*, *IAA28*), two PIN transporters (*PIN1* and *PIN2*), three isopentenyltransferases (*IPT1*, *IPT2* and *IPT5*; involved in cytokinin biosynthesis) and two lipoxygenases (*LOX1* and *LOX2;* involved in JA biosynthesis) were selected for expression analysis. ABA also mediates LR inhibition through ABI4^17^. Although ABI3 (a B3 domain containing TF) fine tunes the auxin-mediated LR formation, it works in close association with ABI4 (an AP2 TF) along with ABI5 in many ABA-dependent signaling pathways^18^,^19^. Hence, these three genes were also considered. As ethylene also inhibits lateral root formation^20^, five well characterized ERFs (*RAP2.2*, *RAP2.3*, *RAP2.12*, *HRE1* and *TINY2*) were also added to the list of genes for transcript analysis.

**Figure 5:**
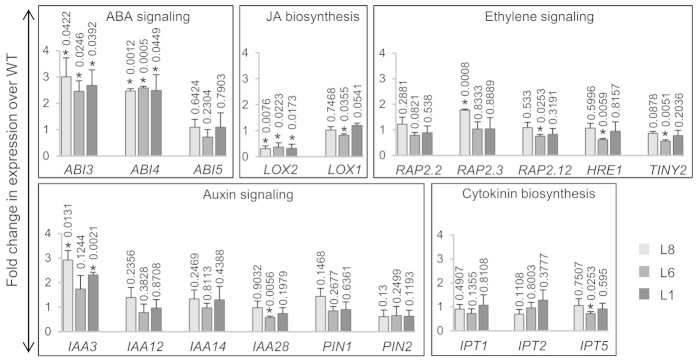
Fold-change in expression of different genes in transgenics (L8, L6 and L1) over WT plants. The analyzed genes and the respective hormonal pathways are mentioned for each histogram. The error bars represent the SD. ‘*’ indicates data significantly different from WT (*t*-test, two-tailed p-value ≤ 0.05). The p-value is mentioned over the respective bars of the histograms**.** The analysis was performed with 15-day old seedlings grown on MS medium in three independent batches (biological replicates). For each independent set of experiment, the sampling was done from a single Petri plate supporting the growth all transgenic and WT plants.

To ensure robustness of the data, transcript level of these genes were monitored in three different transgenic lines (L1, L6 and L8) and results were considered significant only if all these lines exhibited similar trend compared to WT. *ABI3*, *ABI4* and *IAA3* transcripts were enriched by 2 or more folds in the transgenic plants compared to WT (Figure 5). About 3-fold downregulation of *LOX2* was also observed in these transgenic lines. Coexpression analysis (using ‘Abiotic stress’ option; correlation coefficient cut off 0.5) in Rice Oligonucleotide array database (ROAD) also shows that expression of *OsTCP19* positively correlates with nine *IAAs*, thirteen *AP2* (like *ABI4*) and one B3 domain protein (like ABI3). In addition, a negative correlation between *OsTCP19* and a *LOX* gene (similar to *Arabidopsis* *LOX2*) was observed (Supplementary Table S2 online). This further suggests that *OsTCP19* negatively influences auxin and JA signaling, and affects expression of similar class of genes in *Arabidopsis* and rice. Presence of Class-I TCP binding sites (site-II elements) in the promoter of many of these genes points to a possibility of direct regulation by OsTCP19 (Supplementary Fig. S6a online).

### *OsTCP19* influences lipid droplet synthesis and metabolism

**Figure 6:**
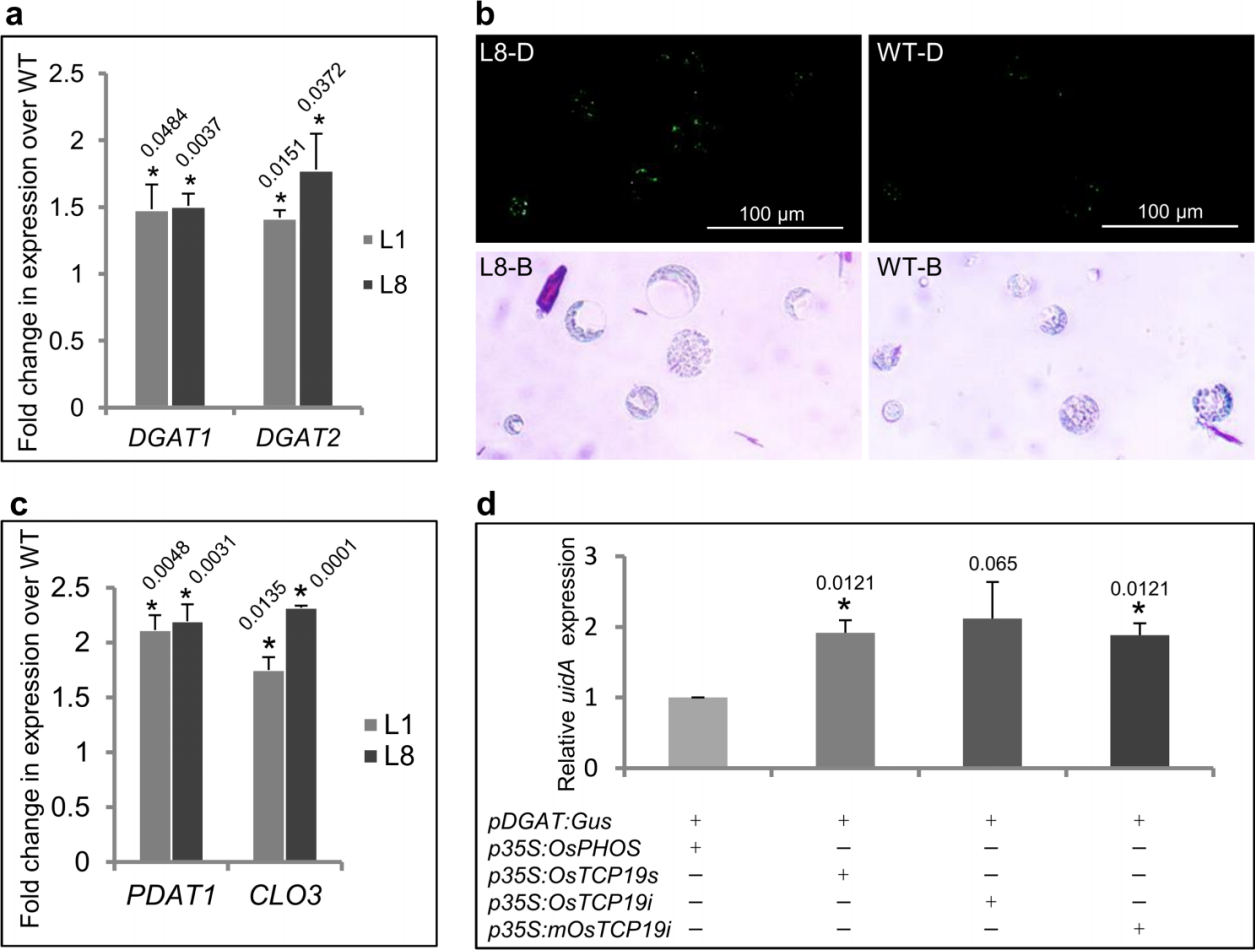
*OsTCP19* influences LD accumulation. (a,c) Fold-change in expression of *AtDGAT1,* *AtDGAT2, PDAT1 and CLO3* in *p35S:OsTCP19* (L1 and L8) over WT *Arabidopsis* plants. (b) Fluorescence microscopy images of Nile red stained LDs in leaf protoplasts of *transgenic* (L8-D) and wild type (WT-D) plants. The image of the same protoplasts under bright field is also shown (L8-B and WT-B). (d) Expression analysis of *uidA* gene in tobacco leaves transiently transformed with constructs as indicated. Error bar in the histograms represents SD and ‘*’ indicates data significantly different from the respective controls (*t*-test, two-tailed p-value ≤ 0.05). The p-value is mentioned over the respective bars of the histograms. All analyses were performed with 15-day old seedlings grown on MS medium in three independent batches (biological replicates). For each independent set of experiment, the sampling was done from a single Petri plate supporting the growth all transgenic and WT plants.

Under abiotic stress, upregulation of triacylglycerol (TAG) biosynthesis gene *diacylglycerolacetyl transferase (DGAT1)* by ABI4 leads to accumulation of lipid droplets (LDs) in vegetative tissue of *Arabidopsis*^21^. Incidentally, *dgat1 Arabidopsis* mutants are hypersensitive to abiotic stresses during seedling establishment which is in contrast to that observed in case of *p35S:OsTCP19* *Arabidopsis* transgenics^22^. By qRT-PCR analysis, about 1.5 times higher expression of *DGAT1* in the transgenic (L1 and L8) than WT plants was observed (Figure 6a) and appears to be consistent with the increase in *ABI4* expression. Nile red staining of leaf protoplasts revealed hyperaccumulation of LDs in transgenic line L8 (relative to WT; Figure 6b). Further analysis revealed increased expression of two other stress-responsive TAG biosynthesis genes^21^, *DGAT2* and *phospholipid:diacylglycerol acyltransferase 1 ****(****PDAT1*), in transgenic lines by *ca.* 1.5-fold and 2-fold, respectively (Figure 6a,c). A LD-associated *Arabidopsis* protein Caleosin 3 (CLO3 or RD20; a peroxygenase) is known to play an important role in abiotic stress signaling^23^. Analysis in ROAD suggests positive correlation between expression of *OsTCP19* and two caleosin genes, one of them (*LOC_Os03g12230*) being highly similar to *Arabidopsis* *CLO3* (Supplementary Table S2 online). Higher expression of this gene was also observed in *p35S:OsTCP19* transgenics compared to WT *Arabidopsis* plants (Figure 6c).

The *OsDGAT* gene (*LOC_Os02g48350*) was observed to coexpress with *OsTCP19* in ROAD (Supplementary Table S2 online). Its promoter also contains three distinct Class-I TCP TF binding sites (Supplementary Fig. S6b online). Using *promoter:GUS* construct bearing 1097 bp DNA region upstream of start codon of this gene (*pOsDGAT:uidA*) and effector constructs prepared using ORF encoding *OsTCP19s* activation of *OsDGAT* expression by OsTCP19 was demonstrated by agroinfiltration of tobacco leaves. Construct bearing a rice gene encoding a member of secretory phosphatases (*p35S:OsPHOS*; *LOC_Os01g57240*) and unrelated to nuclear activities was used as control effector. Nearly 2-fold upregulation of *uidA* was inferred by qRT-PCR analysis for leaf zones co-expressing *OsTCP19s* compared to those co-expressing the control effector (Figure 6d). In this analysis, the plant selection marker of these vectors, *hptII*, was used as reference gene. OsTCP19i and mOsTCP19i also caused activation of *OsDGAT* to essentially similar levels (Figure 6d). All these data indicate that *OsTCP19* plays an important role in stress signaling by influencing LD biosynthesis as well as its metabolism.

### OsTCP19 interacts with OsABI4 and OsULT1

**Figure 7:**
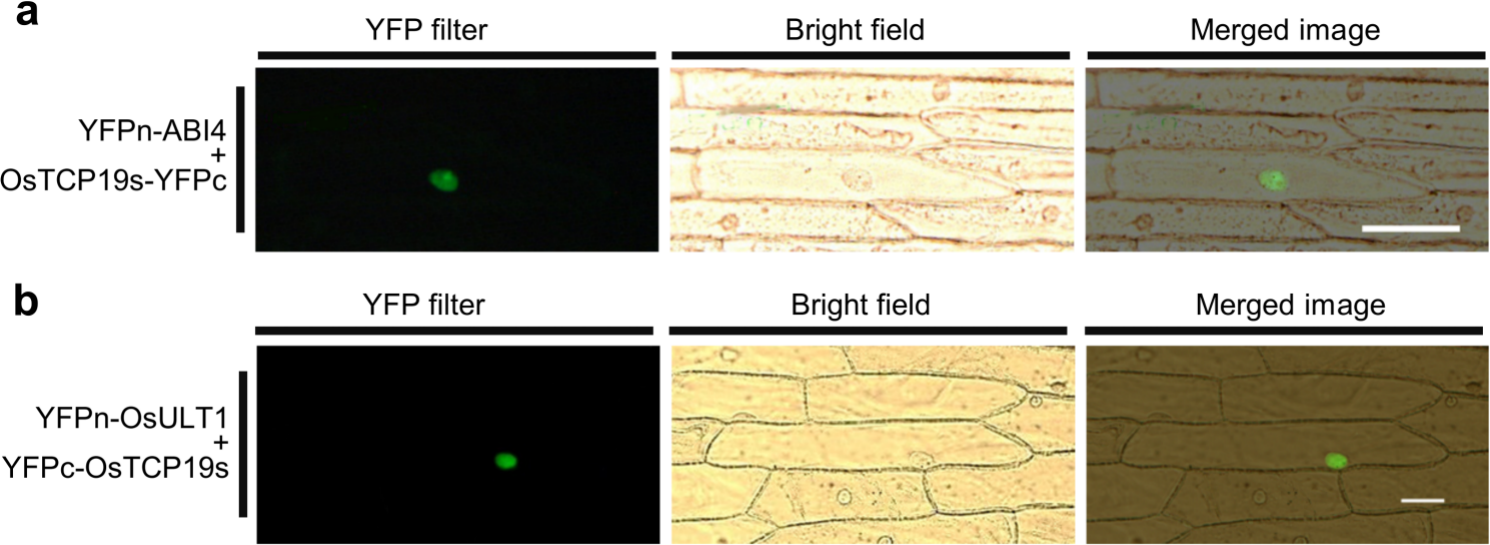
BiFC analysis showing nucleus specific interaction of OsTCP19 with OsABI4 and OsULT1. (a) Fluorescence microscopy images of onion epidermal cells cotransfected with *p35S:YFPn-OsABI4* and *p35S:OsTCP19s-YFPc* constructs (scale bar = 100 µm). (b) Similar analysis for cells cotransfected with *p35S:YFPn-OsULT*1 and *p35S:YFPc-OsTCP19s* (scale bar = 50 µm).

*p35S:OsTCP19* *Arabidopsis* transgenics showed better seedling establishment and survival under abiotic stresses and conditional early flowering which contradict the established activities of the upregulated genes *ABI3* and *ABI4*^21^,^24^,^25^. This led to hypothesize a model involving condition-dependent regulation of *ABI3* and *ABI4* beyond transcriptional level in the transgenic plants and might involve physical interaction of these proteins with OsTCP19. A bimolecular fluorescence complementation (BiFC) assay was carried out to test this hypothesis in rice using constructs bearing N-terminus of *YFP* fused to N-terminus of *OsABI4* (*LOC_Os05g28350*; *p35S:YFPn-OsABI4*) and C-terminus of YFP fused to C-terminus of *OsTCP19s* (*p35S:OsTCP19s-YFPc*). On particle bombardment, YFP fluorescence was detected exclusively in nucleus of the transformed onion epidermal cells. Thus, it indicated a nucleus-specific interaction between OsTCP19 and OsABI4 (Figure 7a). This also substantiates the hypothesis that OsTCP19 can modulate the activity of OsABI4 and control pathways in rice as observed in *Arabidopsis*.

A subset of NB localizing proteins in metazoans is known to contain SAND-domain^26^. OsTCP19 was envisaged to interact with few such proteins based on the presence SAND-domain containing proteins in plants and localization of OsTCP19 to NBs. Two functionally redundant SAND-domain containing transcriptional regulators, ULT1 and ULT2, regulate set of genes including those belonging to KNOX1 group which, interestingly, are also the targets of many Class-I TCP proteins^5^,^27^. This further suggests a possibility of interaction between OsTCP19 and ULT-like genes from rice. BiFC analysis in onion epidermal cells using constructs bearing N-terminus *YFP* fused to N-terminus of *OsULT1* (*LOC_Os01g57240*; *p35S:YFPn-OsULT1*) and C-terminus YFP fused to N-terminus of *OsTCP19s* (*p35S:YFPc-OsTCP19s***)** revealed strong YFP fluorescence in the nucleus (Figure 7b). Thus, OsTCP19 can interact with ULT-like proteins which are known coregulators of transcription. Similar analyses also indicate interaction of OsABI4 and OsULT1 with the protein encoded by the unspliced form of OsTCP19 (Supplementary Fig. S7, S8 online). No true fluorescence was observed when BiFC assays were done for various negative controls (Supplementary Fig. S9 online). Interaction of OsABI4 and OsULT1 with OsTCP19s was also observed in yeast two-hybrid assays, thus substantiating BiFC data (Supplementary Fig. S10 online).

## Discussion

Nuclear localization of TCP proteins is expected as they belong to TF family. Besides whole nucleus, OsTCP19 was also localized in NBs. Apart from general eukaryotic NBs (like speckles, histone locus body, stress granules etc.)^28^, those containing cyclophillin, HYL1, phytochrome and AKIP1 are known to exist in plants^29^. However, the nature of NBs where OsTCP19s and OsTCP19i localize remains to be determined.

ULT1 and ULT2 are trithorax group (trxG) factors^30^ and function by recruiting trxG proteins like ATX1, which is a histone H3 lysine 4 tri-methyltransferase and regulator of dehydration response in *Arabidopsis*^31^. TrxG proteins antagonise the activity of polycomb group (PcG) gene repression complexes which include MSI1, a negative regulator of stress signaling and Class-I TCP regulated pathways^12^. Hence, the interaction between OsTCP19 and OsULT1 might suggest the existence of a regulatory module comprised of few Class-I TCP TFs, ULT1- and ATX1-like proteins that can configure the abiotic stress signal network.

The decrease in LR number in *p35S:OsTCP19* *Arabidopsis* transgenic plants were attributed to upregulation of *ABI4* and *IAA3* and downregulation of *LOX2.* These expression changes could even explain slow initial root growth and increased formation of trichomes and RHs in the transgenics^32^,^33^,^34^. Reduction in LR number is considered as an adaptive response towards drought stress tolerance^35^. RHs bear membrane integrated H^+^-ATPases which mediate root-to-shoot signaling and maintain osmoregulation and water content under drought stress^36^. Trichomes aid in reducing transpiration and behave as a sink for glutathione which contributes in combating oxidative stress and water loss under harsh environmental conditions^37^. Thus, the phenotypes displayed by *p35S:OsTCP19* transgenics also contribute to abiotic stress tolerance. Earlier, it has been found that *TCP14*/*15* regulate trichome formation and disturbances in TCP20 activity severely affect roots elongation in *Arabidopsis*^38^,^39^.

Earlier studies reveal better drought tolerance for mutants of jasmonate signaling in plants^40^. Jasmonate signaling promotes ROS production^41^ which though aids in generating stress responses but is deleterious to plants at higher concentration^42^. Hence, by regulating *LOX2* expression, *OsTCP19* might assist in keeping a partial check over ROS concentration. This in turn provides an optimum chance for survival under environmental stresses. Upregulation of *IAA3* in the transgenic plants might have negated the effect of increased expression of *ABI3* which usually has a role in auxin-mediated LR formation^14^,^19^. Interestingly, *OsTCP19* upregulates under cold stress (as per microarray data) and *ABI3* also provides freezing tolerance in *Arabidopsis*^43^. Few Class-I TCPs are known to regulate the expression of *IAA3* (e.g. AtTCP15) positively and *LOX2* negatively (e.g. AtTCP20) by binding to their promoter sequences in *Arabidopsis*^3^,^44^. Presence of Class-I TCP binding sites in upstream region of similar genes which are positively or negatively coexpressed with *OsTCP19* imply the conservation of similar regulatory pathways in rice.

Knowledge about the role of LDs in vegetative tissues is limited. Recent studies have shown hyperaccumulation of LDs in vegetative tissues of *Arabidopsis* in response to various abiotic stresses and hormones treatments^21^. Similar accumulation of TAG has also been reported for monocots under abiotic stress^45^. Despite a positive correlation between the increase in LDs and inhibition of seedling establishment, LDs are still considered to have a role in seed germination and seedling establishment under various stresses. This is evident from *Arabidopsis* *dgat1* mutant plants which are compromised in these traits^22^. Elevated expression of DGAT1 during both cell division (in shoot and root apical meristem) and senescence (of leaves) suggests a contrasting role for LD in these processes^46^,^47^. Thus, it is being hypothesized that by serving as a rich source of energy and nutrients to sustain cell division, increased levels of TAG in *p35S:OsTCP19* *Arabidopsis* plants might support seedling establishment under conditions severely affecting normal metabolic pathways, like abiotic stresses.

*OsTCP19* caused hyperaccumulation of LDs by increasing the expression of multiple abiotic stress-upregulated TAG biosynthesis genes like *DGAT1*, *DGAT2* and *PDAT1*, and this is partly dependent on ABI4 as it directly activates *DGAT1* in association with ABI5^21^. In the present study, two-fold increase in the activity of *OsDGAT* promoter due to direct activation by OsTCP19 was also observed. A higher activation might depend on relative abundance of other endogenous factors which probably were limiting during the transient assays in tobacco leaves. An earlier study also reported the failure of many Class-I TCP proteins from rice to cause any transactivation in cultured tobacco cells or mesophyll protoplasts by co-transfection assays^1^. In another case, AtTCP20-EAR (AtTCP20 fused to EAR repression domain) failed to repress PCNA in *Arabidopsis* transgenics, although binding of AtTCP20 to PCNA promoter has been shown by *in vitro* and *in vivo* assays^38^.

Recent studies revealed that LDs *per se* could be of little importance and, in fact, the associated proteins and the process of lipid metabolism decide their function^48^. Expression of *CLO3* was increased in *p35S:OsTCP19* *Arabidopsis* plants. This Ca^2+^-binding LD-associated caleosin protein upregulates in vegetative tissue under abiotic stresses and plays a role in drought tolerance by reducing transpiration^23^. The expression of a similar gene from rice also correlates (as per ROAD) with that of *OsTCP19*. Similar *CLO3*-regulated pathways in rice may get affected by *OsTCP19*.

In the present study, early flowering of the transgenics in vertically oriented Petri plates revealed the condition-dependent activity of *OsTCP19*. Earlier studies reported condition-dependent contrasting functions for TCP14 and TCP15 in *Arabidopsis*^49^. This study postulates a conditional regulation of ABI4 activity by OsTCP19 and was substantiated by the interaction between these proteins. This hypothesis seems to fit in a model that will allow increased tolerance to dehydration and salinity, and phenotype like early flowering to occur despite higher expression of *ABI4* and *ABI3*. Interestingly, mutants of *TCP14* show hypersensitivity to ABA during germination and expression of a dominant repressor form of TCP15 affects seedling establishment in *Arabidopsis*^44^,^50^. Although, genes of similar classes were found to coexpress with *OsTCP19* in rice, none were direct homologue of *ABI3* or *ABI4*. However, a conditional upregulation of these genes by OsTCP19 remains possible due to disturbances in other hormone (like auxin) pathways^17^.

Based on many reports, ABI4 was chosen as a target of direct regulation by OsTCP19. First, *ABI4* is known to regulate *ABI3* expression in many signaling pathways^18^. Second, ABI4 is a dynamic transcription factor which can switch its activity from activator to repressor in a conditional manner^51^. Third, it can serve as a link between cytokinin and ABA signaling^17^. Coincidently, Class-I TCP TFs also play a role in cytokinin-dependent pathways and, in the present study too, *OsTCP19* transcript levels were upregulated by exogenous ABA application. Moreover, ABI4 from monocots are functionally similar to that from *Arabidopsis*^52^,^53^,^54^. These reports and the present results together suggest a role for OsTCP19 in fine-tuning ABA signaling by regulating the expression and activity of key proteins like ABI4.

In short, the present study assigns a role for *OsTCP19* in calibrating and crosslinking the developmental and stress-response pathways by interfering with auxin and JA acid pathways and manipulation of the ABA-signaling network. It could partly mediate this by recruiting trxG factor for activation of the target genes and by interacting with key regulators like ABI4. Its role in stress tolerance is mediated by the accumulation of LDs and associated proteins besides reduction in cell death, water loss and ROS production. The phenotypes displayed by the transgenics, stress tolerance assays and expression analysis together indicate an extensive role of *OsTCP19* in water-deficit stress signaling. However, it might be involved in shaping the early signaling pathways in response to various abiotic stresses. In conclusion, this study unravels the role of a rice gene, *OsTCP19* and extends the role of Class-I TCP TFs in abiotic stress response and ABA signaling.

## Methods

### Plant material and growth conditions

Sterilized (70% ethanol, 1 min; 3.5% NaOCl, 40 min) seeds of PB1, Pokkali and N22 indica rice were grown in liquid Yoshida medium^55^ under 12 h light condition. 10-day-old seedlings were subjected to different treatments (as mentioned in results) in hydroponic culture system in the presence of Yoshida medium. Sterilized (70% ethanol, 30 sec; 0.6% NaOCl and 0.001% Tween-20, 10 min) *Arabidopsis thaliana* Col-0 seeds were germinated and grown on Murashige and Skoog (MS; Duchefa) medium containing 1% sucrose and 0.8% purified agar under continuous illumination at 21^o^C following stratification. For salt and water-deficit treatments, NaCl (100 mM or 125 mM) or mannitol (200 mM or 350 mM) were provided as additives to *Arabidopsis* growing medium. When required, 6-8 leaf stage, healthy plants were transferred to pots containing Soilrite (a combination of Vermiculture, Perlite and Spagnum moss; 1:1:1 ratio) and irrigated with RO water. During assessment of stress tolerance in pots, either irrigation was stopped or was done with 200 mM NaCl solution every four days. For recovery from these stresses, plant were irrigated with normal RO water and observed for one week. Samples were either processed for further analysis or stored under frozen condition till use. All experiments were done with at least three biological replicates.

### *In* *silico* analysis

Details about the databases and software used for doing various *in silico* analyses are mentioned in ‘Supplementary Table S3 online’.

### Gene expression analysis

RNA was extracted from different samples using Trizol (Sigma). cDNA was synthesized using ‘Applied biosystems High capacity cDNA synthesis kit’ (Life technologies). Expression analysis of genes or their splice forms was achieved either by qRT-PCR following manufacturer’s protocol (‘Applied biosystems 7500 fast real time machine’ and ‘Applied biosystems fast SYBR green mix’, Life technologies) or semi-qRT-PCR under standard PCR conditions followed by gel electrophoresis. Sequences of all primers used for the analyses are mentioned in ‘Supplementary Table S4 online’. For qRT-PCR analysis either rice *Ubiquitin5* (*UBQ5*; for rice samples) or *Arabidopsis* *Actin2* (*ACT2*; for *Arabidopsis* samples) were used as reference genes. The Ct values obtained for various samples were first normalized with that for the respective reference gene (ΔCt). To obtain fold change in expression (as per the case), the ΔCt values of various genes for different samples were again normalized to that for unstressed tissue (0 h samples) or the wild type plants (ΔΔCt). The final values for fold change in expression were derived by calculating 2^–ΔΔCt^. To compare the abundance of *OsTCP19* transcripts across various rice varieties, 2^–ΔCt^ were calculated which represent the relative expression level of the gene with respect to the reference gene.

### Preparation of different constructs

*OsTCP19* was cloned in TA-cloning vector (pGEMT-easy, Promega) following its amplification by PCR using PB1 rice genomic DNA and primers flanking the ORF (Supplementary Table S5 online, S.No. 1-2). By incorporating *Nco*I and *Spe*I sites as overhangs in the concerned primers (Supplementary Table S5 online, S.No. 3-4), the ORF with its stop codon was PCR amplified from *OsTCP19_PGEMT* plasmid and mobilized into pCAMBIA1302 vector (www.cambia.org) between CaMV *35S* promoter and mGFP using the facility of these restriction sites to create *p35S:OsTCP19* construct. Applying ‘Phusion site directed mutagenesis kit’ (Thermo scientific) and primers as described in ‘Supplementary Table S5 online (S.No. 7-8)’, a mutated version of *OsTCP19* (*mOsTCP19i*) was created by replacing the first GC and last AG dinucleotides of the intron to GG and AA, respectively. *OsABI4* (*LOC_Os05g28350*; homologous to *ABI4* from *Zea mays* and *Arabidopsis*), *OsULT1* (*LOC_Os01g57240*; homologous to *Arabidopsis* *ULT1* and *ULT2*) and spliced *OsTCP19* form were amplified from PB1 cDNA using primer as mentioned in Supplementary Table S5 online (S.No. 1-2, 9-10, 13-14). As per manufacturer’s instructions, primers were designed (Supplementary Table S5 online, S.No. 5-6, 11-12, 15-16) and following PCR the ORFs of *OsULT1*, *OsABI4* and *OsTCP19* (spliced, unspliced and mutated forms) were first cloned in pENTR-D-Topo entry vector (Invitrogen, Life Technologies) and then mobilized into various destination vectors (Supplementary Table S6 online, S.No. 1-13) by recombination using ‘Invitrogen LR clonase II mix’ (Life technologies). Similarly, 1097 bp genomic fragment upstream of *OsDGAT* (*LOC_Os02g48350*) was also amplified and cloned in Gateway vector for the preparation of *pOsDGAT:uidA* construct (Supplementary Table S5 online, S.No 17-20; Supplementary Table S6 online, S.No. 17).

### Yeast Two-Hybrid analysis

*OsABI4* and *OsULT1* were cloned in PGBKT7-DEST (bait vector) and *OsTCP19s* in PGADT7-DEST (prey vector) by gateway cloning to create *OsABI4-BD*, *OsULT1-BD* and *OsTCP19-AD,* respectively (Supplementary Table S6 online, S.No 14-16). Pairwise co-transformation of these constructs into *Saccharomyces cerevisiae* AH109 cells was conducted using EZ-transformation kit (MP biomedical) and were then selected and grown on appropriate medium to check their interactions as per BD Matchmaker protocol (Clontech).

### Subcellular localization and BiFC analysis

For subcellular localization, construct *p35S:YFP-OsTCP19s* or *p35S:YFP-mOsTCP19i* was bombarded on onion epidermal cells as described^56^ and imaged by fluorescence microscopy (Eclipse 80i, Nikon). For BiFC, any of the *p35S:OsTCP19s-YFPc*, *p35S:OsTCP19i-YFPc*, *p35S-mOsYFP19i-YFPc*, *p35S:YFPc-OsTCP19s*, *p35S:YFPc-OsTCP19i* or *p35S:YFPc-mOsTCP19i* construct was co-expressed in onion epidermal cells by particle bombardment and visualized by fluorescence or confocal microscopy (AOBS TCS-SP2, Leica). Experiments were validated from at least three separate sets of bombardment, each done with four different onion peels.

### *Agrobacterium*-mediated *Arabidopsis* transformation

*Arabidopsis* plants transformation was done using *Agrobacterium tumefaciens* strain GV3101 bearing *p35S:OsTCP19* construct by floral dip method as described by Giri *et al*^56^*.* Transgenic selection was done on hygromycin (15 mg/ml) containing medium. During selection of T_1_ plants, a plant line negative for hygromycin resistance was selected and maintained as a negative control plant (NT).

### Visualization of oil bodies

Protoplasts isolated from leaves of 15-day-old plants^57^ were stained for 10 min with 0.1% Nile red (stock solution in acetone) in MMG buffer followed by two brief washings. The protoplasts were then visualized by fluorescence microscopy (Nikon 80i) using FITC filter.

### Analysis of abiotic stress-related parameters

To examine post-germination biomass accumulation of seedlings, the ratio for the total weight of seedlings developed from hundred seeds under stress and control condition was calculated. Measurements of relative water content (RWC) and the analysis of cell death by Evan’s blue staining in the leaves of Arabidopsis plants were done according to Ji *et al.*^58^. The percentage water loss measurements from excised leaves were done according to Saez *et al.*^59^. ROS accumulation, was studied by staining leaves with 100 µM 2′,7′-dichlorodihydrofluorescein diacetate (H_2_DCFDA) in 10 mM Tris-HCl, pH 7.2 and imaging the fluorescing stomata using a fluorescence microscope. Fluorescence signal from more than fifty stomata per leaf were quantified in ImageJ software for preparing a graphic representation of the data. Data presented are average of three replicates in each case.

### Agroinfiltration of tobacco leaves

This was achieved by injecting a mix of equal proportion of *Agrobacterium tumefaciens* strain LBA4404 bearing *pDGAT:uidA* construct and those bearing either *p35S:OsPHOS*, *p35S:OsTCP19s*, *p35S:OsTCP19i* or *p35S:OsmTCP19i* into tobacco leaves as described by Pandey *et al*^60^*.* Each analysis was performed in leaves from three different plants.

## Author Contributions

**Authors contributions** PM and AKT conceived and designed the experiments, and analyzed all the data. PM performed all the experiments, wrote the initial manuscript draft and prepared all the figures. AKT did final corrections in the manuscript.

## Supplementary Material

Supplementary Information Supplementary information
