# Supplementary material for: OsTCP19 influences developmental and abiotic stress signaling by modulating ABI4-mediated pathways
Source: Sci Rep. 2015 Apr 29;5:9998. doi: 10.1038/srep09998 (PMC4415230; doi:10.1038/srep09998)
Supplement: Supplementary Information [file srep09998-s1.pdf]

TITLE: *OsTCP19* influences developmental and abiotic stress signaling by modulating ABI4-mediated pathways

AUTHORS: Pradipto Mukhopadhyay (**e-mail:** pradiptom@gmail.com)
Akhilesh Kumar Tyagi (**e-mail:** akhilesh@genomeindia.org)

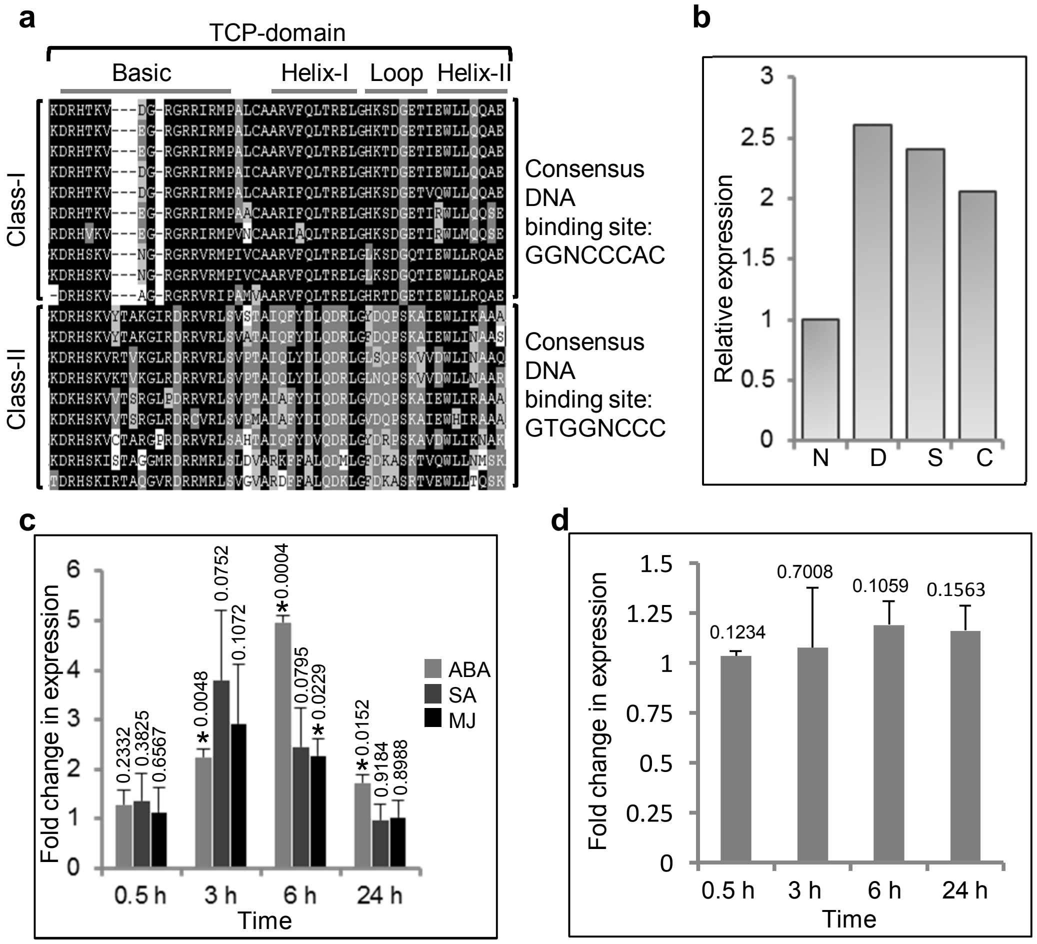

Figure S1 | A rice Class-I TCP TF, *OsTCP19*, is upregulated under abiotic stress. (a) Sequence feature of the TCP-domain of Class-I and Class-II rice TCP TFs. (b) Microarray (GSE6901) analysis showing relative expression of *OsTCP19* under control (N) dehydration (D) salt (S) and cold (C) stress in rice seedlings according to normalized data obtained from Rice Oligonucleotide Array Database (ROAD). (c) qRT-PCR analysis depicting fold-change in expression of *OsTCP19* following ABA (Sigma, Cat. No. A1049) , salicylic acid (SA; Sigma, Cat no. S5922) and methyl jasmonate (MJ; Aldrich, Cat no. 392707) treatments in PB1 rice shoot over control (untreated; 0 h sample; value equivalent to 1) samples. (d) Similar qRT-PCR analysis of PB1 shoot samples for seedlings incubated in Yoshida medium for different time periods. Error bars represent SD and * indicates significantly different data from control (*t*-test, two-tailed p-value ≤ 0.05). The p-value is mentioned over the respective bars. For qRT-PCR analyses, the data were simulated from three independent sets of experiments.

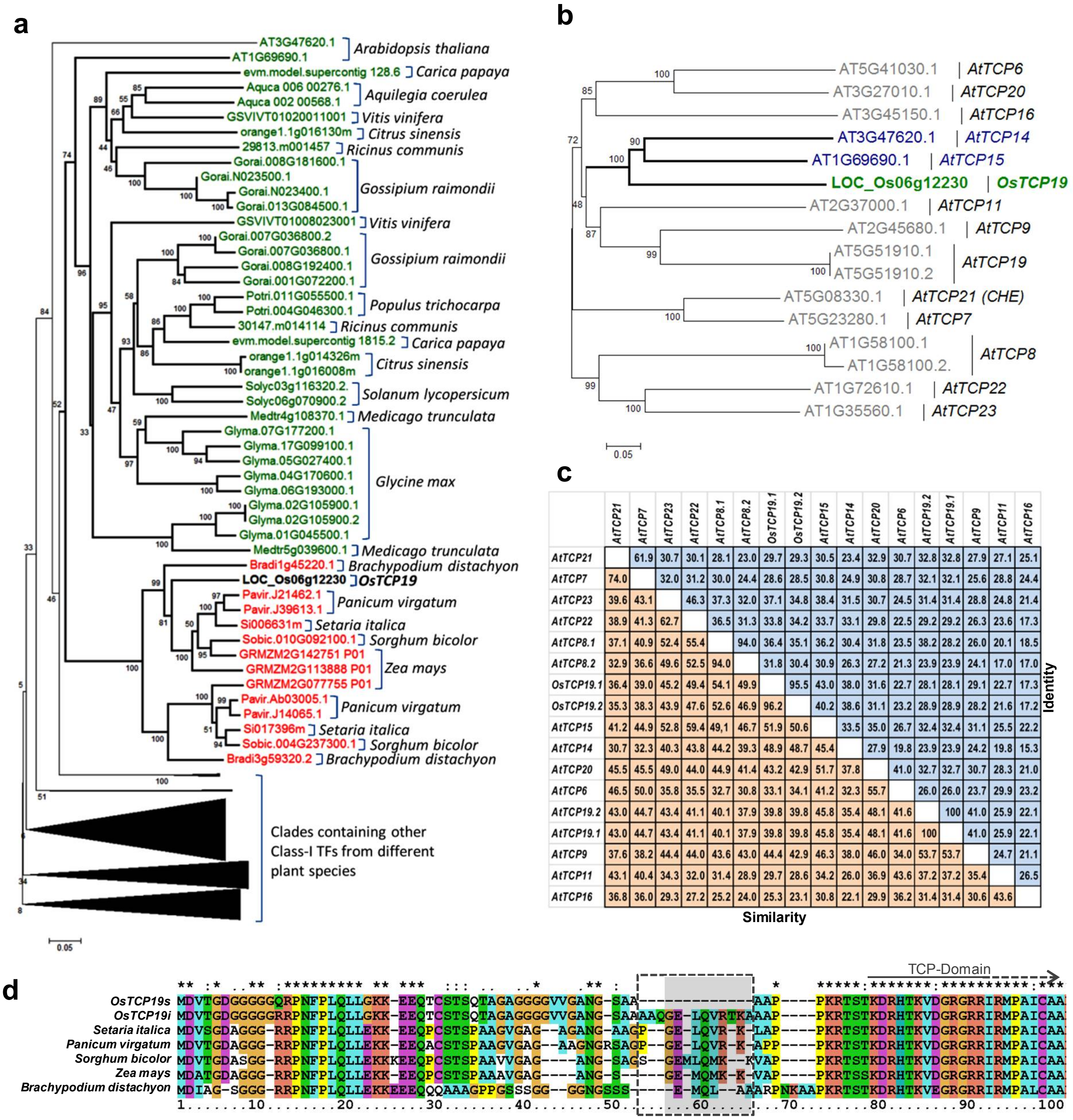

Figure S2 | Phylogenetic analysis of OsTCP19. (a) Bootstrap neighbour-joining tree created by MEGA5 software showing similarity of different Class-I TCP TF sequences from various plants to OsTCP19 following ClustalW alignment. All sequences were obtained by a BLASTp search in Phytozome v10 database using OsTCP19 sequence as query. The clade represented by bold lines contains sequences that are highly similar to OsTCP19. The dicot and monocot protein ids have been coded with green and red colors, respectively. Other sequences having lesser similarity to OsTCP19 form separate clades (outgroups) that have been compressed and are represented by black triangles. The gene ids of all these sequences are mentioned in supplementary Table S1 online. (b) Similar analysis specifically showing homology of OsTCP19 to the Class-I TCP proteins of *Arabidopsis thaliana.* (c) MatGAT2.02 derived pairwise similarity/identity matrix between Class-I TCP TFs and OsTCP19 encoded by spliced (*OsTCP19.1*) and unspliced (*OsTCP19.2*) forms. (d) ClustalW alignment showing the region (marked by a box) and amino acid stretch (shaded portion) similar to that encoded by the intron of *OsTCP19* in N-terminus portion of homologous proteins from other monocot plants.

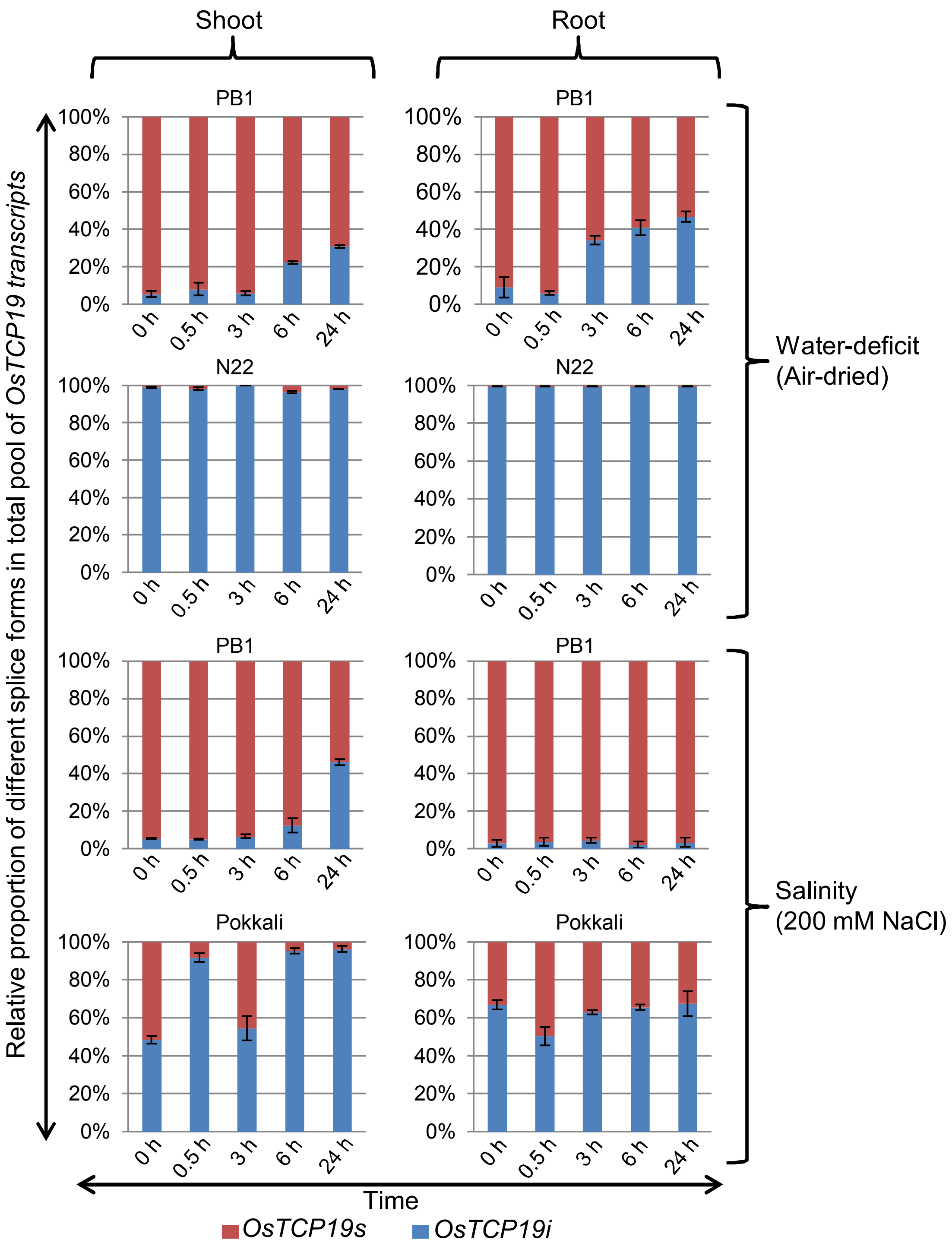

Figure S3 | Abundance of different splice forms of *OsTCP19* in indica rice varieties under various conditions. RT-PCR products obtained for unstressed (0 h) and stressed (0.5-24 h) tissues of PB1, N22 and Pokkali rice seedlings (as indicated) using 36-i primers were quantified by imageJ software and graphical plots were made to represent the proportion of *OsTCP19* spliced forms under various condition. For calculation, background intensity was deducted from each reading. The error bars represent SD. The data was simulated from three independent set of experiments.

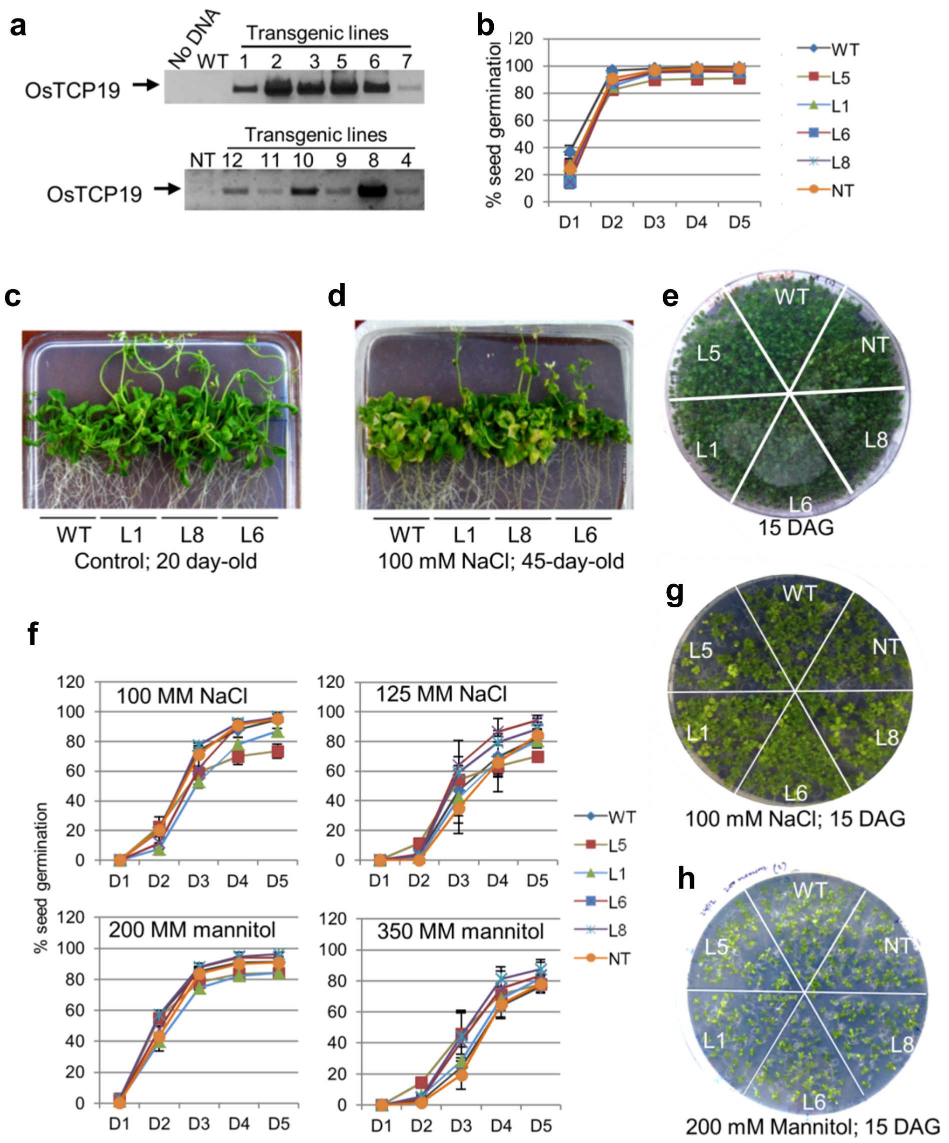

Figure S4 | Comparison of phenotypes of non-transgenic and *OsTCP19* over-expressing transgenic plants at various stages and conditions. (a) Tissue-PCR confirmation of T0 transgenic plants. (b,f) Germination rate and efficiency of WT, NT and transgenic lines under control and stress conditions (as indicated). (c,d) Early flowering of transgenic plants under control and mild stress conditions. (e,g,h) Post-germination growth and seedling establishment in case of various plant lines under control and mild stress conditions (as indicated). The error bars corresponds to SD. All data were simulated from three independent set of experiments.

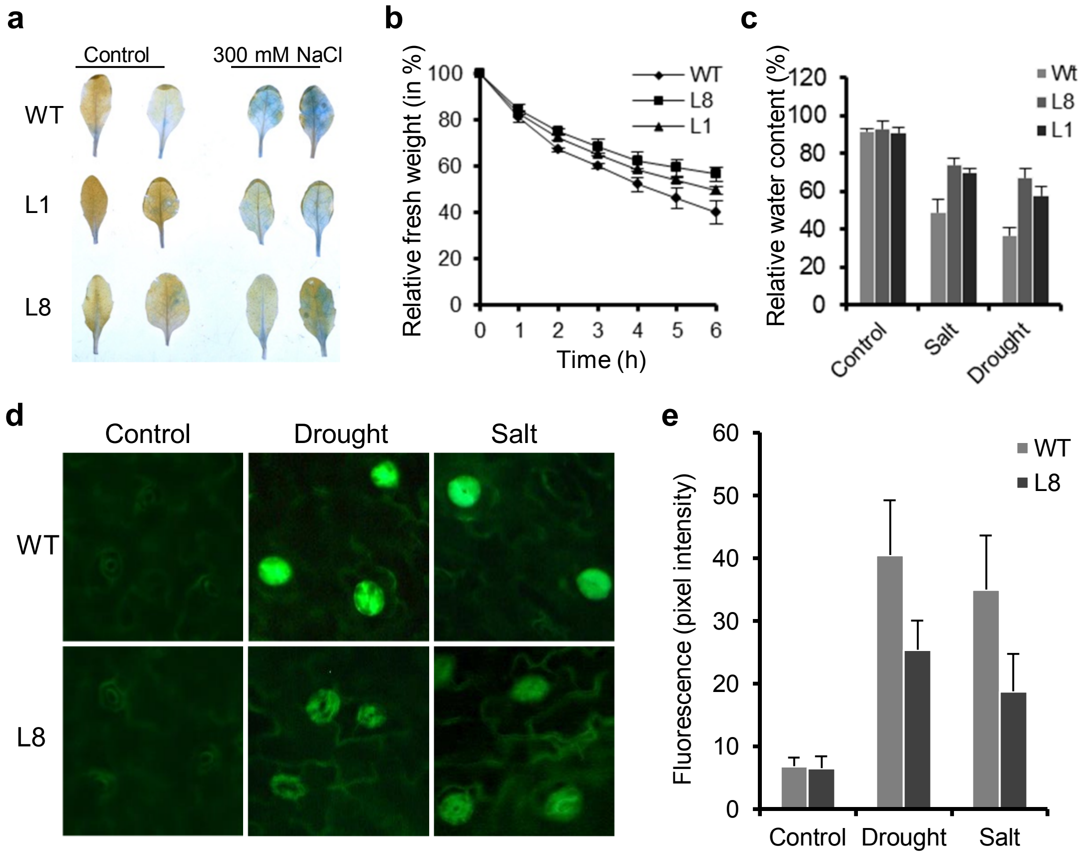

Figure S5 | Reduction in cell death, water loss and ROS accumulation under stress in *p35S:OsTCP19 Arabidopsis* plants. (a) Evans blue staining of detached leaves from 22-day-old plants that were incubated (15 h) in water or salt solution. (b) Time-course analysis of water loss from detached leaves of 22-day-old plants when kept under air drying. (c) %RWC measurements from leaves of 24-day-old plants subjected to salt (200 mM NaCl) or water-deficit stress for 12 days. (d) Fluorescence microscope images showing H_2DCFDA stained leaves from 24-day-old plants subjected to 12 days of various stresses as indicated. (e) Quantification of H_2DCFDA florescence using ImageJ sofware. The name of the plant lines used in various analysis is indicated in each figure. Error bars in the graphs represent SD. All analysis were done from leaves of 15 to 30 plants grown in three independent batches.

a

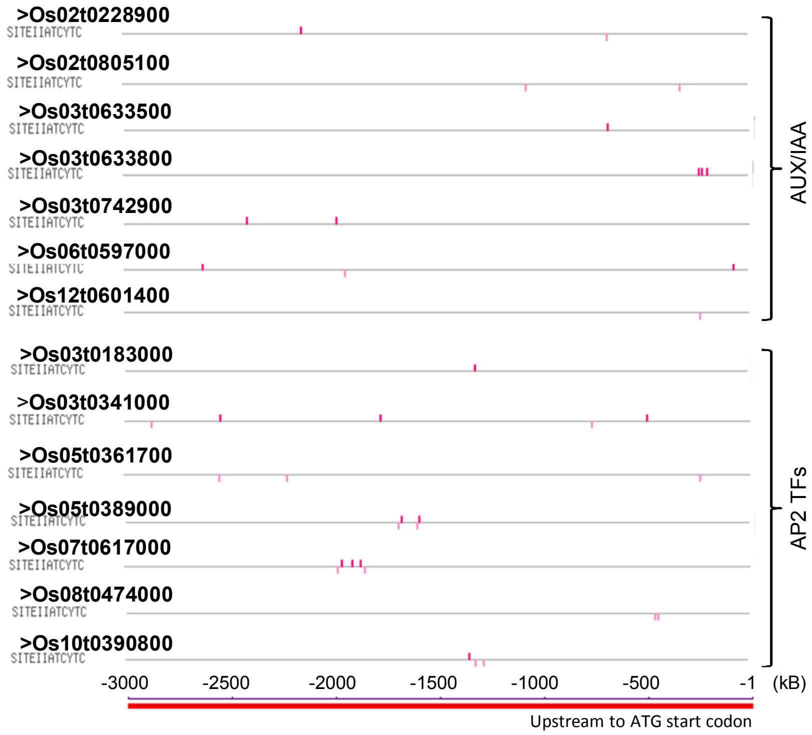

b

```
>>>>>>>>>>>>>>>>>>>
gatgcatgtcactcaatacggtatcagaaccatatatctcaagtatgaatctaggttggc
acacaattaatcaaaaaaaaatacaatccacttctattccacgtgtggagattatgagta
ctatatacccacatagttggtttatcggactggttataggggatattttatatctctata
atatgtaacggattaagacttgggcattatgtttccttaagtattaaaggaaacagccta
caatcctggattcagaatattgtaaaatatatgttgtatttagttaacttttatatgtaa
tcctgccttcctgacctatataagatgggtagtaggccccccttaaggatataaccacact
taagatcatcagatcaatacaacacccgacaggtagaaatctcaaacaacacccgacaag
tagaaatctcaaacatgagaatggtgttacctattgacaagggcctaaactttttataatt
tttttgcaattcaatccatctactttagctctcgtgcagtatgccttcttgtattgccga
ttagtagcatcgacaccatggctgagatatgagggagccttctcatgtaactggatgtat
tgaaatatattagtgaattgtccgtctttttttagtaacttccatttttttattggaatggt
acgtaactcaacagagttatttatatctagttgactccgttaacgtgtactacaacaaca
tacagtaccatagtaacaaagacaccaaacgcaaccgctctagaaagatgagcggtcacc
tcggcttacttacgcaagtgaagtactactacgactaatacttcccttcttcctaccttt
ccctacctgctctgctctctctcacgaacacagagaatcattccccgcacatcgaatccc
acactcaccgacgcgtgggccccaatcactcccgcacatcactgacacgtgggccccacg
cacccagagccagagcccagaaaggcctcgtcctcctcctcctctctgattcgtcaccgc
caccgccgcgtcgtcccgcgtctctcctcacctctcgtctccctcgcgacgtctcccctt
ctccctccacccctccaATG
<<<<<<<<<<<<<<<<<<
```

>>>> Forward primer region ; <<<< Reverse primer region ;
Class-I TCP Binding site ; Similar to TCP binding site;
gtc…… 5′ UTR region ; ATG ATG start codon

Figure S6 | Presence of site-II elements (TCP-binding site) in *OsTCP19* co-expressing genes. (a) Analysis of 3 kb region upstream of genes using PlantPAN. The mentioned gene ids belong to RAP-DB database. (b) Putative promoter region of *OsDGAT* showing TCP protein binding sites, 5’UTR region and primer sequences used for cloning of this region.

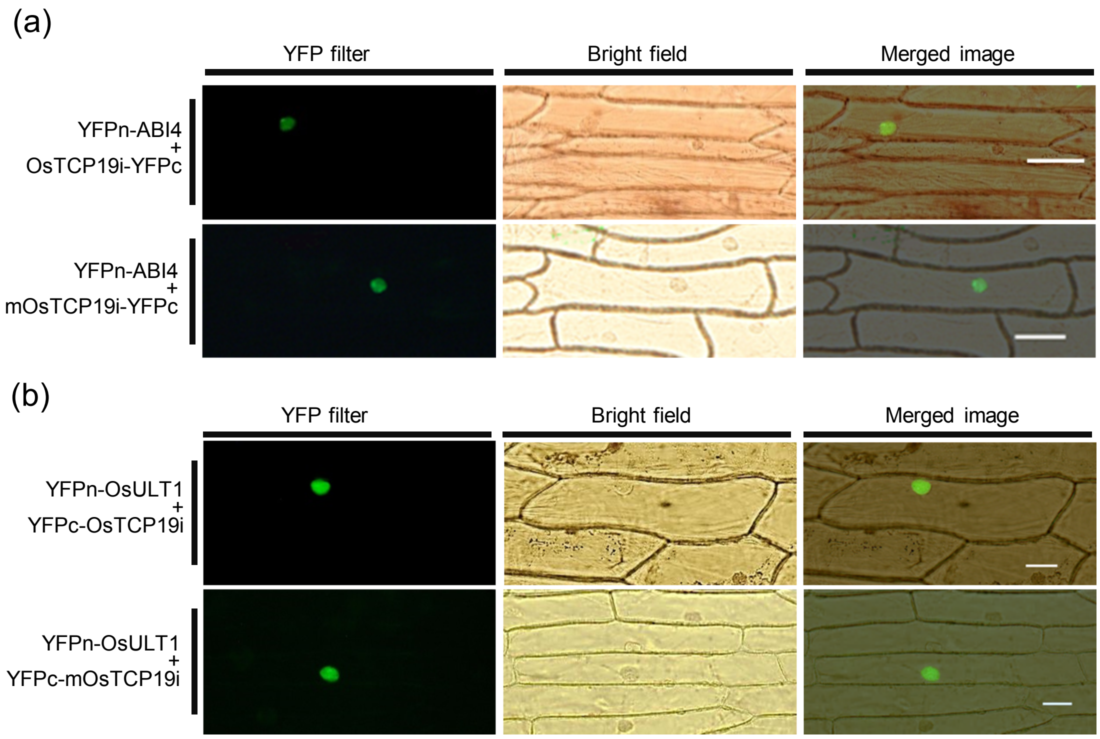

Figure S7 | BiFC analysis showing interaction of OsTCP19 with OsABI4 and OsULT1. (a) Fluorescence microscopy images of onion epidermal cells showing YFP fluorescence in nucleus when *p35S:YFPn-OsABI4* was co-bombarded with *p35S:OsTCP19i-YFPc or p35S:mOsTCP19i-YFP*c (scale bar = 100 µm). (b) Nuclear fluorescence obtained from similar analysis when *p35S:YFPn-OsULT*1 was co-bombarded with *p35S:YFPc-OsTCP19i* or *p35S:YFPc-mOsTCP19i* (scale bar = 50 µm).

(a)

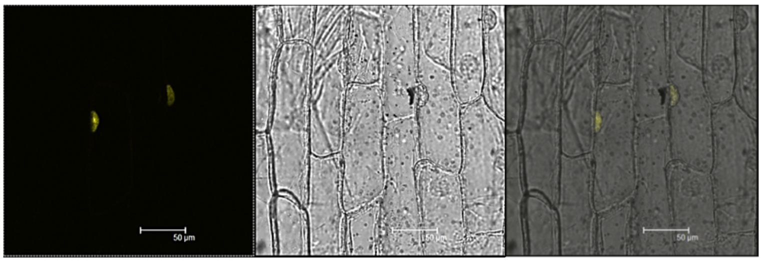

(b)

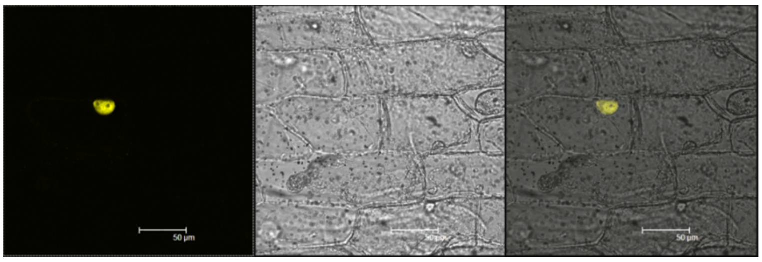

Figure S8 | Confocal laser microscopy images revealing mOsTCP19i interaction with OsABI4 and OsULT1 inside nucleus. (a) BiFC analysis in onion epidermal cells using *p35S:mOsTCPi-YFPc* and *35S:YFPn-OsABI4* constructs. (b) Similar analysis using *p35S:YFPc-mOsTCP19i* and *p35S:YFPn-ULT1* constructs (scale bar = 50 µm).

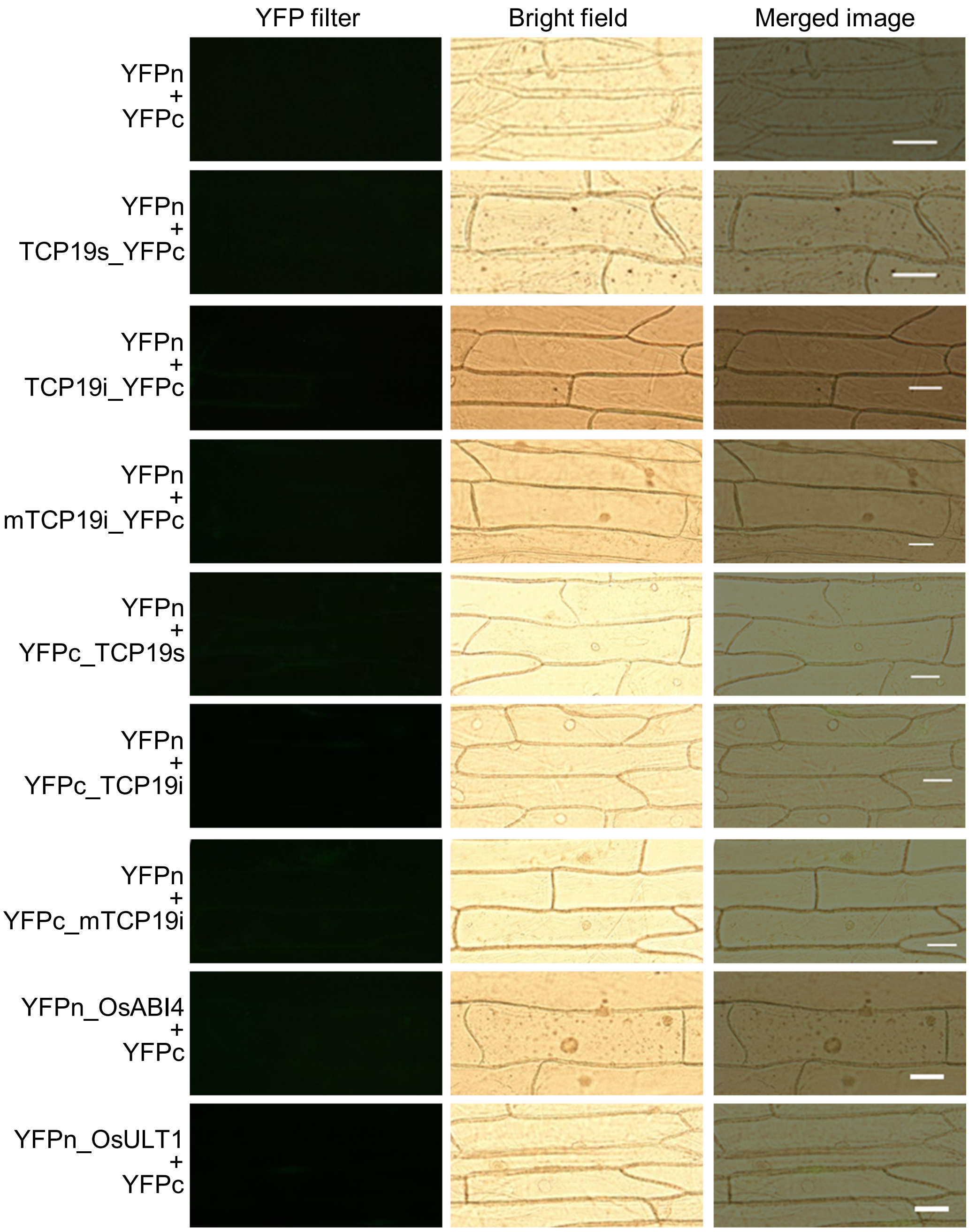

Figure S9 | Negative controls used in BIFC experiments and their respective YFP signals. Different combination of constructs used as negative controls in BiFC experiment is mentioned along with a representative image indicating the obtained degree of fluorescence (scale bar = 50 µm).

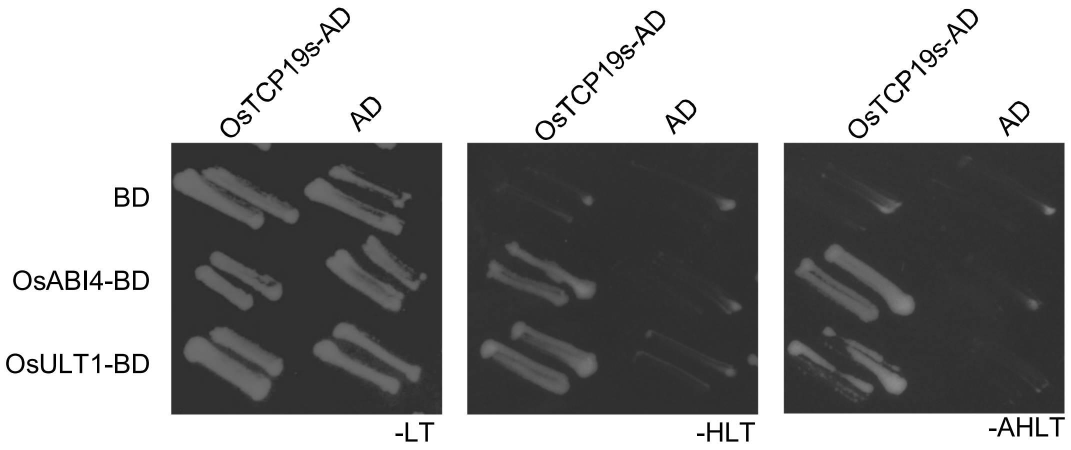

Figure S10 | Yeast two-hybrid analysis showing interaction of OsTCP19 with OsULT1 and OsABI4. The left panel image shows growth of the yeast AH109 cells bearing different combination of constructs in SD medium devoid of leucine and tryptophan (-LT) indicating the presence of both bait and prey vectors in the cells. Middle or right panel images shows growth of the yeast cells growing on SD medium lacking leucine, tryptophan and histidine (-HLT) or lacking leucine, tryptophan, histidine and adenine (-AHLT) indicating the positive interaction between bait and prey constructs. Construct combinations AD:BD, BD:OsTCP19s-AD, OsULT1-BD:AD OsABI4-BD:AD were used as negative controls.

Table S1. List of sequences represented by gene ids which were considered for creating the phylogenetic tree shown in the Supplementary Figure S1a

S.No.	Plant Name	Selected Class-I TCP sequences showing similarity to OsTCP19 with e-value>1e-23 in BLASTp Search
1	***Aquilegia coerulea***	Aquca_002_00568.1, Aquca_006_00276.1, Aquca_011_00080.1, Aquca_013_00460.1, Aquca_014_00153.1, Aquca_021_00091.1, Aquca_021_00091.2, Aquca_021_00091.3, Aquca_030_00105.1, Aquca_035_00256.1, Aquca_091_00069.1
2	***Arabidopsis thaliana***	AT1G35560.1, AT1G58100.1, AT1G69690.1, AT1G72010.1, AT2G37000.1, AT2G45680.1, AT3G27010.1, AT3G47620.1, AT5G08330.1, AT5G23280.1, AT5G41030.1, AT5G51910.1, AT5G51910.2
3	***Brachypodium distachyon***	Bradi1g45220.1, Bradi2g59240.1, Bradi3g49660.1, Bradi3g59320.2, Bradi4g24550.2, Bradi4g35520.1, Bradi4g35670.1, Bradi4g41430.1, Bradi5g02880.1, Bradi5g16270.1
4	***Carica papaya***	evm.model.supercontig_128.6, evm.model.supercontig_18.176, evm.model.supercontig_1815.2, evm.model.supercontig_30.48, evm.model.supercontig_4.210, evm.model.supercontig_5.261, evm.model.supercontig_60.75, evm.model.supercontig_66.26, evm.model.supercontig_70.118
5	***Citrus sinensis***	orange1.1g008187m, orange1.1g014326m, orange1.1g016008m, orange1.1g016130m, orange1.1g021068m, orange1.1g021100m, orange1.1g021111m, orange1.1g023883m, orange1.1g024547m, orange1.1g048727m
6	***Glycine max***	Glyma.01G045500.1, Glyma.02G105900.1, Glyma.02G105900.2, Glyma.03G018800.1, Glyma.04G170600.1, Glyma.05G027400.1, Glyma.05G050400.1, Glyma.06G193000.1, Glyma.07G080300.1, Glyma.07G177200.1, Glyma.08G299400.1, Glyma.08G299400.2, Glyma.09G284300.1, Glyma.09G284500.1, Glyma.10G057400.1, Glyma.10G240200.1, Glyma.10G285900.1, Glyma.11G196000.1, Glyma.12G168300.1, Glyma.12G168300.2, Glyma.13G144100.1, Glyma.16G004300.1, Glyma.16G053900.1, Glyma.17G099100.1, Glyma.17G132400.1, Glyma.18G121400.1, Glyma.18G121400.2, Glyma.19G095300.1, Glyma.20G001600.1, Glyma.20G103400.1, Glyma.20G154400.1, Glyma.20G154400.2
7	***Gossypium raimondii***	Gorai.001G072200.1, Gorai.001G273300.1, Gorai.001G273300.2, Gorai.002G215000.1, Gorai.002G215000.2, Gorai.002G215000.3, Gorai.004G206900.1, Gorai.005G211900.1, Gorai.005G211900.2, Gorai.005G211900.3, Gorai.006G043800.1, Gorai.006G165300.1, Gorai.006G197000.1, Gorai.007G036800.1, Gorai.007G036800.2, Gorai.007G094200.1, Gorai.008G147800.1, Gorai.008G157300.1, Gorai.008G157300.2, Gorai.008G181600.1, Gorai.008G192400.1, Gorai.008G243000.1, Gorai.009G289000.1, Gorai.011G086900.1, Gorai.012G084600.1, Gorai.012G084600.2, Gorai.012G084600.3, Gorai.012G166500.1, Gorai.013G068600.1, Gorai.013G084500.1, Gorai.013G268200.1, Gorai.N023400.1, Gorai.N023500.1
8	***Medicago truncatula***	Medtr1g038650.1, Medtr1g038650.2, Medtr1g063870.1, Medtr1g101810.1, Medtr1g101810.2, Medtr1g101810.3, Medtr1g101810.4, Medtr1g114380.1, Medtr2g006150.1, Medtr2g006150.2, Medtr4g108370.1, Medtr5g039600.1, Medtr7g028160.1, Medtr8g033070.1
9	***Panicum virgatum***	Pavir.Aa01128.1, Pavir.Aa01258.1, Pavir.Ab03005.1, Pavir.Ba01284.1, Pavir.Ca00220.1, Pavir.Ea03897.1, Pavir.Fa00111.1, Pavir.Ga00835.1, Pavir.Ga02405.1, Pavir.Ha00851.1, Pavir.J00025.1, Pavir.J03131.1, Pavir.J05134.1, Pavir.J12088.1, Pavir.J14065.1, Pavir.J15424.1, Pavir.J20261.1, Pavir.J20370.1, Pavir.J20370.2, Pavir.J21462.1, Pavir.J23534.1, Pavir.J39613.1
10	***Physcomitrella patens***	Phpat.003G097300.1, Phpat.010G055200.1
11	***Populus trichocarpa***	Potri.001G060000.1, Potri.001G060000.2, Potri.001G111800.1, Potri.001G327100.1, Potri.002G152200.1, Potri.003G120200.1, Potri.003G167900.1, Potri.004G046300.1, Potri.004G222100.1, Potri.005G090300.1, Potri.005G090300.2, Potri.005G090300.3, Potri.006G125800.1, Potri.009G009400.1, Potri.011G055500.1, Potri.012G135900.1, Potri.013G110700.1, Potri.013G110700.2, Potri.014G078500.1, Potri.015G138200.1, Potri.016G094800.1, Potri.019G081800.1, Potri.019G081800.2, Potri.T044100.1, Potri.T044100.2
12	***Ricinus communis***	29638.m000529, 29728.m000800, 29813.m001457, 29929.m004612, 30014.m000446, 30078.m002308, 30093.m000364, 30131.m007038, 30147.m014114, 30147.m014152, 30174.m008944
13	***Selaginella moellendorffii***	estExt_fgenesh2_pg.C_150226, estExt_fgenesh2_pg.C_20339, fgenesh2_pg.C_scaffold_2000049
14	***Setaria italica***	Si006631m, Si010765m, Si011076m, Si017396m, Si026501m, Si030591m
15	***Solanum lycopersicum***	Solyc00g084870.2, Solyc01g008230.2, Solyc01g103780.2, Solyc02g068200.1, Solyc02g094290.1, Solyc03g006800.1, Solyc03g116320.2, Solyc04g009180.1, Solyc05g007420.1, Solyc06g065190.1, Solyc06g070900.2, Solyc08g080150.1, Solyc09g008030.1, Solyc11g020670.1
16	***Sorghum bicolor***	Sobic.002G268600.1, Sobic.003G408400.1, Sobic.004G225400.1, Sobic.004G237300.1, Sobic.004G354700.1, Sobic.005G059000.1, Sobic.006G025000.1, Sobic.006G154000.1, Sobic.010G092100.1
17	***Vitis vinifera***	GSVIVT01008023001, GSVIVT01019876001, GSVIVT01020011001, GSVIVT01023283001, GSVIVT01026145001, GSVIVT01027588001
18	***Zea mays***	GRMZM2G003944_P01, GRMZM2G034638_P01, GRMZM2G089638_P01, GRMZM2G092214_P01, GRMZM2G092214_P02, GRMZM2G093895_P01, GRMZM2G096610_P01, GRMZM2G107031_P01, GRMZM2G113888_P01, GRMZM2G142751_P01, GRMZM2G178603_P01, GRMZM2G416524_P01, GRMZM2G445944_P01, GRMZM2G465091_P01

Table S2. Partial list of genes co-expressing positively (S.No. 1-26) and negatively (S.no. 27) with *OsTCP19* as determined in ROAD

S.No.	Gene id	Corr. Co-eff.	Gene Description	Gene Family
1	LOC_Os03g43410	0.6406	OsIAA12 - Auxin-responsive Aux/IAA gene	AUX/IAA
2	LOC_Os12g40890	0.5964	OsIAA30 - Auxin-responsive Aux/IAA gene	
3	LOC_Os12g40900	0.589	OsIAA31 - Auxin-responsive Aux/IAA gene	
4	LOC_Os03g43400	0.5887	OsIAA11 - Auxin-responsive Aux/IAA gene	
5	LOC_Os06g39590	0.5719	OsIAA23 - Auxin-responsive Aux/IAA gene	
6	LOC_Os03g53150	0.5494	OsIAA13 - Auxin-responsive Aux/IAA gene	
7	LOC_Os02g13520	0.5311	OsIAA7 - Auxin-responsive Aux/IAA gene f......	
8	LOC_Os02g56120	0.5049	OsIAA9 - Auxin-responsive Aux/IAA gene f......	
9	LOC_Os01g13030	0.5045	OsIAA3 - Auxin-responsive Aux/IAA gene f......	
10	LOC_Os03g08470	0.7133	AP2 domain containing protein, expressed	AP2/ERF
11	LOC_Os07g42510	0.6536	AP2 domain containing protein, expressed	
12	LOC_Os05g32270	0.5831	AP2 domain containing protein, expressed	
13	LOC_Os02g43970	0.5548	AP2 domain containing protein, expressed	
14	LOC_Os05g29810	0.546	AP2 domain containing protein, expressed	
15	LOC_Os04g52090	0.5427	AP2 domain containing protein, expressed	
16	LOC_Os02g54160	0.5323	AP2 domain containing protein, expressed	
17	LOC_Os08g36920	0.5221	AP2 domain containing protein, expressed	
18	LOC_Os03g22170	0.5182	AP2 domain containing protein, expressed	
19	LOC_Os10g25170	0.5067	AP2 domain containing protein, expressed	
20	LOC_Os02g13710	0.5846	ethylene-responsive transcription factor......	
21	LOC_Os03g09170	0.5822	ethylene-responsive transcription factor......	
22	LOC_Os06g36490	0.5022	ethylene-responsive element-binding prot......	
23	LOC_Os01g04750	0.5436	B3 DNA binding domain containing protein......	B3-domain
24	LOC_Os03g12230	0.5903	caleosin related protein, putative, expr......	Caleosin
25	LOC_Os06g14370	0.529	caleosin related protein, putative, expr......	
26	LOC_Os02g48350	0.5832	diacylglycerol O-acyltransferase, putati......	DGAT
**27**	**LOC_Os02g10120**	**-0.5679**	**lipoxygenase, putative, expressed**	**LOX**

Table S3. List of databases and softwares used for various *in silico* analysis

S.No.	Software/Database Name	Acronym	Web site (if online)	Purpose
1	National Centre for Biotechnology Information	NCBI	www.ncbi.nlm.nih.gov	Sequence retrieval / BLAST search
2	Rice Genome Annotation Project	RGAP	rice.plantbiology.msu.edu	Rice Sequence retrieval / BLAST search
3	The Rice Annotation Project Database	RAP-DB	rapdb.dna.affrc.go.jp	Rice Sequence retrieval / BLAST search / Gene id conversion
4	Phytozome v9.1	—	www.phytozome.net	Sequence retrieval / Blast search
5	The Arabidopsis Information Resource	TAIR	www.arabidopsis.org	Arabidopsis Sequence retrieval / Blast search
6	EMBOSS suite	—	—	All general sequence analysis
7	ClustalW	—	—	Sequence alignment / N-J Phylogenetic tree file generation
8	Genedoc	—	—	Editing/refinement of sequence alignments
9	MEGA 5	—	—	Final generation and refinement of phylogenetic tree
10	Oligo Explorer	—	—	Primers designing for DNA cloning and semi-qRT-PCR
11	Primer express	—	—	Primers designing for qRT-PCR
12	Microsoft excel	—	—	All numerical data analysis and graph preparation
13	in-silico	—	in-silico.net/	Statistical analyses of various data
14	GIMP	—	—	Editing of Images
15	ImageJ	—	—	Measurements and analysis within an image
16	Rice Oligonucleotide Array Database	ROAD	www.ricearray.org	Retreival of rice microarray data and co-expression analysis
17	PlantPAN		plantpan.mbc.nctu.edu.tw	Analysis of promoter elements

Table S4. Primers used for qRT- and semi-qRT-PCR analysis of various genes and splice forms

S.No.	Forward Primers		Reverse Primers	
	Primer Name	Sequence (5'…3')	Primer Name	Sequence (5'…3')
1	OsTCP19_RT_F	TTTTCTCCGTTTTTGTTTGAGTTG	OsTCP19_RT_R	CATGAATATATGATGGGTCGAGGAA
2	OsUBQ5_RT_F	ACCACTTCGACCGCCACTACT	OsUBQ5_RT_R	ACGCCTAAGCCTGCTGGTT
3	RAP2.3_F	GGCTGAGGAACTCTGGTCAGA	RAP2.3_R	GGTGGAATAGAAACCCCAGAAGT
4	RAP2.2_F	GAGGAAAACCCAGTGGAGCTATG	RAP2.2_R	ACTTCAAAAGTCTCCTTCCAGCAT
5	RAP2.12_F	AATGGGACGCTTCACTGGAT	RAP2.12_R	GCACCATTGTCCTGAGTCGTT
6	HRE1_F	CGCCTAGTAGTAGCTGTCTTGATTTC	HRE1_R	ATTCGCATCGCCCATGAT
8	TINY2_F	ATTCCCTCGACCCGTTTCAT	TINY2_R	CGGTTCCATGTGAGCTGCTT
9	IAA12_F	CCCTGTTTAGCTTCCCTTCCA	IAA12_R	TATCGAGTAAATTGCAAACCTCAAA
10	IAA14_F	CATGAGAGGCGTAGGGAACAA	IAA14_R	TGAAGAGGATGGACTGAATTTGAA
11	IAA14_s2_F	ATGAGAGGCGTAGGGAACAA	IAA14_s2_R	CATTAGCATGAAGAGGATGGACT
12	IAA3_F	TCTCTGTCTGTGCTTGGGTTGT	IAA3_R	CCGATGAGGTAAATAAAGGCTATGA
13	IAA28_F	CTCCTCCTTGTCACCAATTCACT	IAA28_R	TTTGGTCGAGCTTTTTTGTTTAGA
14	PIN1_F	AAGGATCCAGAAGGGAAGATTATTC	PIN1_R	GAGACAATATCTTCACGGATCGATT
15	PIN2_F	TCTACGCAATGTTTAACGCA	PIN2_R	CGCCTTTAGAAGACTGAAGTGA
16	ABI3_F	AACCAATACGCATCAGGAAACTG	ABI3_R	ACCTTGACTGCTGTCTAATGGAATC
17	ABI4_F	CAAAGGTGGTCCGGACAACT	ABI4_R	ACCCATTTGCCCCAGCTT
18	ABI5_F	GGAAGAGGAAGCAACAGTATTTTGA	ABI5_R	CGCAATCTCCCGTTCGATT
19	IPT1_F	CCGGTGGATCTAACTCTTTCGT	IPT1_R	CGAACTTTGGGTCGAATCGT
20	IPT2_F	TGGAATGCGCAAGTGGTTAA	IPT2_R	TTCGGTTTCTGTCTCCAGGAA
21	IPT5_F	GTTCGCCGCATCTTCGAT	IPT5_R	AATGGCTCGGCGAATTCC
22	LOX1_F	CCGGAAATCATCAGCTTATTAGC	LOX1_R	CAATCACAAACGGTTCAATCGAT
23	LOX2_F	CATTTCCGCTACACCATGGA	LOX2_R	CCACCTCCGTTGACAAGACTTT
24	DGAT1_F	GGAGAATGTGGAATATGCCTGTT	DGAT1_R	GCGCAAGCACGGGAAGTA
25	DGAT2_F	CGTGTCGACAGCCTATGCAT	DGAT2_R	TGGCTTCAGAGTTTTTGTGACTTC
26	PDAT1_F	CGCACATGGGACTCAACAAT	PDAT1_R	CCGCCCCATATCGTGTCA
27	CLO3_F	GCCAAAGAGAGGGCCAATTC	CLO3_R	TTATTCGCTAACCAAACACACACA
28	ACT2_RT_F	TGCCAATCTACGAGGGTTC	ACT2_RT_R	TCTCTTACAATTTCCCGCTCTG
29	GusA_RT_F	CCGCAAGGAATCGGTCAAT	GusA_RT_R	ACTTGCAAAGTCCCGCTAGTG
30	HptII_RT-F	GGATTTCGGCTCCAACAATG	HptII_RT_R	CATTCAATGACCGCTGTTATGC
31	OsTCP19_splicing_F	CGGGAAGAAGGAGGAGCAGAC	OsTCP19_splicing_R	CTTCGTGTGCCGGTCCTTC
32	OsEF1α_F	TTTCACTCTTGGTGTGAAGCAGAT	OsEF1α_R	GACTTCCTTCACGATTTCATCGTAA

S.No. 1-2: qRT- and semi-qRT-PCR primers of rice genes; **S.No. 3-2**8: qRT-PCR primers of *Arabidopsis* genes; **S. No. 29-30:** qRT-PCR primers for *GusA* expression analysis in Tobacco infiltration assays; **S.No. 31-32:** Semi-qRT-PCR primers for *OsTCP19* splice form analysis.

Table S5. Primers used for cloning and site-directed mutagenesis (SDM) of various DNA fragments

S.No.	Primer Name	Sequence (5'….3')	Comment
1	OsTCP19_full_F	TATACCATGGCGTGAGAGTAG	PCR amplification of *OsTCP19* including parts of 5' and 3' UTRs
2	OsTCP19_full_R	GGAATGCAACTGTATGATCC	
3	OsTCP19_CDS_1302_NcoI_F	ACGAATTCCATGGATGTCACCGGAGAC	PCR Amplification for cloning of *OsTCP19* CDS between NcoI and SpeI site in pCAMBIA1302
4	OsTCP19_CDS_1302_SpeI_R	CTAACTAGTCTACGAGTCGCTGGCGCT	
5	OsTCP19_pENTR_F	CACCATGGATGTCACCGGAGAC	PCR Amplification for cloning of *OsTCP19* CDS in pENTR-D-Topo gateway entry vector
6	OsTCP19_pENTR_R	CGAGTCGCTGGCGCTCAT	
7	OsTCP19_intron_SDM_F	[Phos]AGCTGCAGGTGAGGACGAAAGCGGCGGCGC	5' phosphorylated primers for site directed mutagenesis of intron boundaries in *OsTCP19*
8	OsTCP19_intron_SDM_R	[Phos]CCCCCTGCGCCGCCCCCGCCGACCCATTCG	
9	OsABI4_full_F	CACTCTCTCTCTGCTTCATCTTCTC	PCR amplification of *OsABI4* including parts of 5' and 3' UTRs
10	OsABI4_full_R	AACCCCACCAAACAAACTACG	
11	OsABI4_CDS_PENTR_F	CACCATGGAGCCCAGCGACGAC	PCR Amplification for cloning of *OsABI4* CDS in pENTR-D-Topo gateway entry vector
12	OsABI4_CDS_PENTR_R	TCACTTGAGGAAGAGATCGAA	
13	OsULT1_full_F	GAGTTGTTGCGGTGTGTTTG	PCR amplification of *OsABI4* including parts of 5' and 3' UTRs
14	OsULT1_full_R	CATGCTGGTGTTTCATCCTAC	
15	OsULT1_CDS_pENTR_F	CACCATGGCTGCGGCGGCGAA	PCR Amplification for cloning of *OsULT1* CDS in pENTR-D-Topo gateway entry vector
16	OsULT1_CDS_pENTR_R	CTACTCCTTGGAGTTATGGTAG	
17	pDGAT_1st_PCR_F	TGCATCCATGCTTAGATTCG	PCR amplification of a portion of gDNA containing a 5' region of *OsDGAT* and further upstream region
18	pDGAT_1st_PCR_R	AACGTGAACGCGGAGAAGTC	
19	pDGAT_1.2_PENTR_F	CACCGATGCATGTCACTCAATACG	PCR amplification for clonning of 1.2 kb region upstream of *OsDGAT* ATG codon in pENTR-D-Topo gateway entry vector
20	pDGAT_up_ATG_PENTR_R	TGGAGGGGTGGAGGGAG	

Table S6. List of recombinant clones prepared by Gateway technology and their parental entry and destination vectors

S.No.	Entry clone	Destination vector	Recombinant clone	Destination vector source
1	OsTCP19s_pENTR	pSITE 3CA	p35S:YFP-OsTCP19s	Chakraborty et al. (2007)#
2	OsTCP19s_pENTR	pSAT5A-DEST-c(175-end)EYFP-N1	p35S-OSTCP19s-YFPc	ABRC## Stock - CD3-1096
3	OsTCP19s_pENTR	pSAT5-DEST-c(175-end)EYFP-C1(B)	p35S:YFPc-OsTCP19s	ABRC## Stock - CD3-1097
4	OsTCP19s_pENTR	pMDC32	p35S:OSTCP19s	ABRC## Stock - CD3-738
5	OsTCP19s_pENTR	pGAGT7-DEST	OsTCP19-AD	Horak et al. (2008)###
6	mOsTCP19i_pENTR	pSITE 3CA	p35S:YFP-mOsTCP19i	Chakraborty et al. (2007)#
7	mOsTCP19i_pENTR	pSAT5A-DEST-c(175-end)EYFP-N1	p35S-mOSTCP19i-YFPc	ABRC## Stock - CD3-1096
8	mOsTCP19i_pENTR	pSAT5-DEST-c(175-end)EYFP-C1(B)	p35S:YFPc-mOsTCP19i	ABRC## Stock - CD3-1097
9	mOsTCP19i_pENTR	pMDC32	p35S:mOSTCP19i	ABRC## Stock - CD3-738
10	OsTCP19i_pENTR	pSAT5A-DEST-c(175-end)EYFP-N1	p35S-OSTCP19i-YFPc	ABRC## Stock - CD3-1096
11	OsTCP19i_pENTR	pSAT5A-DEST-c(175-end)EYFP-C1(B)	p35S:YFPc-OsTCP19i	ABRC## Stock - CD3-1097
12	OsTCP19i_pENTR	pMDC32	p35S:OSTCP19i	ABRC## Stock - CD3-738
13	OsABI4_pENTR	pSAT4-DEST-n(1-174)EYFP-C1	p35S:YFPn-OsABI4	ABRC## Stock - CD3-1089
14	OsABI4_pENTR	pGBKT7-DEST	OsABI4-BD	Horak et al. (2008)###
15	OsULT1_pENTR	pSAT4-DEST-n(1-174)EYFP-C1	p35S:YFPn-OsULT1	ABRC## Stock - CD3-1089
16	OsULT1_pENTR	pGBKT7-DEST	OsULT1-BD	Horak et al. (2008)###
17	pDGAT_pENTR	pMDC164	pDGAT:uidA	ABRC## Stock - CD3-756

Chakrabarty, R. et al.((2007) PSITE vectors for stable integration or transient expression of autofluorescent protein fusions in plants: probing Nicotiana benthamiana-virus interactions. Mol Plant Microbe Interact 20, 740-750 (2007).

Arabidopsis Biological Resource Center

Horak, J. et al. (2008) The Arabidopsis thaliana response regulator ARR22 is a putative AHP phospho-histidine phosphatase expressed in the chalaza of developing seeds. BMC Plant Biol 8, 77.
